# SALM4 suppresses excitatory synapse development by *cis*-inhibiting *trans*-synaptic SALM3–LAR adhesion

**DOI:** 10.1038/ncomms12328

**Published:** 2016-08-02

**Authors:** Eunkyung Lie, Ji Seung Ko, Su-Yeon Choi, Junyeop Daniel Roh, Yi Sul Cho, Ran Noh, Doyoun Kim, Yan Li, Hyeyeon Kang, Tae-Yong Choi, Jungyong Nam, Won Mah, Dongmin Lee, Seong-Gyu Lee, Ho Min Kim, Hyun Kim, Se-Young Choi, Ji Won Um, Myoung-Goo Kang, Yong Chul Bae, Jaewon Ko, Eunjoon Kim

**Affiliations:** 1 Department of Biological Sciences, Korea Advanced Institute for Science and Technology, Daejeon 305-701, Korea; 2 Department of Biochemistry, College of Life Science and Biotechnology, Yonsei University, Seoul 120-749, Korea; 3 Center for Synaptic Brain Dysfunctions, Institute for Basic Science, Daejeon 305-701, Korea; 4 Department of Anatomy and Neurobiology, School of Dentistry, Kyungpook National University, Daegu 700-412, Korea; 5 Center for Cognition and Sociality, Institute for Basic Science, Daejeon 305-701, Korea; 6 Department of Physiology and BK21 PLUS Project to Medical Sciences, Yonsei University College of Medicine, Seoul 120-752, Korea; 7 Department of Physiology, Seoul National University School of Dentistry, Seoul 110-749, Korea; 8 Department of Anatomy and Division of Brain Korea 21, Biomedical Science, College of Medicine, Korea University, Seoul 136-705, Korea; 9 Graduate School of Medical Science and Engineering, Korea Advanced Institute for Science and Technology, Daejeon 305-701, Korea

## Abstract

Synaptic adhesion molecules regulate various aspects of synapse development, function and plasticity. These functions mainly involve *trans*-synaptic interactions and positive regulations, whereas *cis*-interactions and negative regulation are less understood. Here we report that SALM4, a member of the SALM/Lrfn family of synaptic adhesion molecules, suppresses excitatory synapse development through *cis* inhibition of SALM3, another SALM family protein with synaptogenic activity. *Salm4*-mutant (*Salm4*^−/−^) mice show increased excitatory synapse numbers in the hippocampus. SALM4 *cis*-interacts with SALM3, inhibits *trans*-synaptic SALM3 interaction with presynaptic LAR family receptor tyrosine phosphatases and suppresses SALM3-dependent presynaptic differentiation. Importantly, deletion of *Salm3* in *Salm4*^−/−^ mice (*Salm3*^−/−^; *Salm4*^−/−^) normalizes the increased excitatory synapse number. These results suggest that SALM4 negatively regulates excitatory synapses via *cis* inhibition of the *trans*-synaptic SALM3–LAR adhesion.

## Abstract (web summary)

Synaptic adhesion molecules regulate synapse development and function by both *cis* and *trans*-interactions. Here, Lie *et al*. show that postsynaptic SALM4 regulates excitatory synapse numbers by *cis* inhibition of the SALM3-LAR transynaptic interaction.

## Graphical abstract

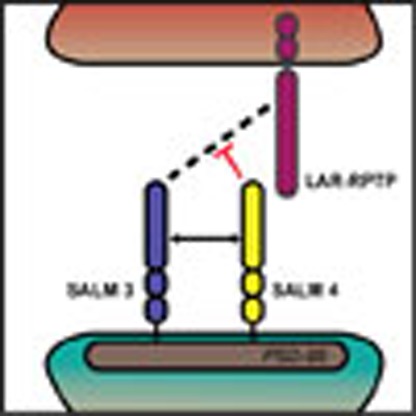

Synaptic adhesion molecules regulate synapse development, function and plasticity. Previous studies have identified a large number of synaptic adhesion molecules, including neuroligins and neurexins^1^,^2^,^3^,^4^,^5^,^6^,^7^,^8^,^9^,^10^,^11^. The known functions of these molecules include aspects of *trans*-synaptic interactions between pre- and postsynaptic adhesion molecules and positive synapse regulations (that is, promotion of synapse development, maturation and maintenance), although opposite aspects (*cis*-interactions between nearby synaptic adhesion molecules on pre- or postsynaptic membranes and negative regulations) are less well understood.

Synaptic cell adhesion-like molecules (SALMs; also known as Lrfns) represent a family of leucine-rich repeat (LRR)-containing synaptic cell adhesion molecules, with five known members: SALM1/Lrfn2, SALM2/Lrfn1, SALM3/Lrfn4, SALM4/Lrfn3 and SALM5/Lrfn5 (refs 12, 13, 14, 15). SALMs share a similar domain structure, containing six LRRs, an immunoglobulin (Ig) domain, a fibronectin III (FNIII) domain in the extracellular region, a transmembrane domain and a cytoplasmic region. Despite these apparent similarities in their overall structures, however, SALMs also possess several structurally distinct features. For instance, their cytoplasmic regions differ substantially in their lengths and amino-acid (aa) sequences. In addition, SALM1–3 (but not SALM4 and -5) contain a C-terminal PDZ-binding motif that binds to the PDZ domains of postsynaptic density (PSD)-95 (refs 12, 13, 14, 15).

Functionally, SALMs regulate neurite outgrowth and branching^12^,^16^,^17^. In addition, they have been implicated in the regulation of synapse development and function. For instance, SALM1 interacts with the GluN1 subunit of NMDA receptors (NMDARs), and promotes dendritic clustering of NMDARs in cultured neurons^12^. SALM2 associates with both NMDARs and AMPA receptors (AMPARs), and promotes the development of excitatory (but not inhibitory) synapses^13^. SALM3 and SALM5 (but not the other SALMs) have synaptogenic activities, promoting both excitatory and inhibitory presynaptic differentiation in contacting axons^18^ via *trans*-synaptic interactions with presynaptic LAR family receptor tyrosine phosphatases^19^. However, with the exception of SALM3, the functions of the SALMs have not been examined using *in vivo* approaches, such as mouse genetics.

Although individual SALM isoforms are structurally and functionally distinct, they have been shown to associate with one another and are likely to form larger complexes^20^. Specifically, SALM1–3 (but not SALM4 and -5) were found to form a co-immunoprecipitable complex in the rat forebrain. In heterologous cells, all of the SALMs can interact with one another, further extending the complexity of inter-SALM interactions^20^. In addition to these *cis*-interactions, SALMs can participate in *trans*-interactions, with SALM4 and -5 forming homophilic and *trans*-cellular adhesion complexes^20^. These results suggest that the individual SALM isoforms are unlikely to be physically or functionally isolated, but instead may participate in intricate interplays with other SALM isoforms. However, the details of such interactions remain largely unexplored.

In the present study, we generated and characterized *Salm4*-mutant (*Salm4*^−/−^) mice. To our surprise, we found that *Salm4*^−/−^ mice display increased excitatory and inhibitory synapse numbers in the hippocampus. Our data suggest that SALM4 *cis*-interacts with SALM3, inhibits the *trans*-synaptic interaction of SALM3 with presynaptic LAR and suppresses SALM3-dependent presynaptic differentiation at excitatory synapses.

## Results

### SALM4 expression pattern in the CNS

**Figure 1:**
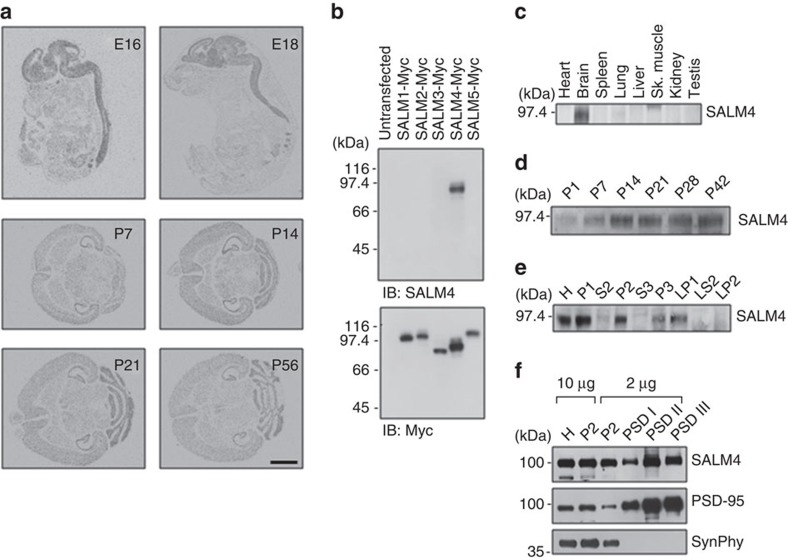
Expression patterns of SALM4 mRNAs and proteins. (**a**) Distribution patterns of SALM4 mRNAs in mouse embryonic (E16 and E18) sagittal sections and postnatal brain (P7, P14, P21 and P56) horizontal sections, as revealed by *in situ* hybridization. E, embryonic day; P, postnatal day. Scale bar, 6 mm. (**b**) SALM4 antibodies specifically recognize SALM4 but not other SALM family proteins. C terminally Myc-tagged SALM family proteins (SALMs 1–5) expressed in HEK293T cells were immunoblotted with SALM4 antibodies (1820 antibody). (**c**) Tissue distribution of SALM4 proteins (1820 antibody). (**d**) SALM4 protein expression increases during postnatal rat brain development (1820 antibody). (**e**) SALM4 protein distribution in rat brain fractions (1820 antibody). H, homogenates; P1, cells and nucleus-enriched pellet; P2, crude synaptosomes; S2, supernatant after P2 precipitation; S3, cytosol; P3, light membranes; LP1, synaptosomal membranes; LS2, synaptosomal cytosol; LP2, synaptic vesicle-enriched fraction. (**f**) Detection of SALM4 proteins in PSD fractions (2620 antibody). PSD-95 and synaptophysin (SynPhy) were used as controls. Note that the prestained markers here seem to migrate slightly faster than normal markers in other panels.

In order to understand the functions of SALM4, we first determined the expression patterns of *Salm4* in the nervous system. SALM4 mRNAs were detected in the mouse brain and spinal cord at embryonic days 16 and 18, as determined by *in situ* hybridization (Fig. 1a). In postnatal brains, SALM4 mRNAs were detected in various brain regions, including the neocortex, striatum and hippocampus.

SALM4 proteins (∼95 kDa) were mainly detected in the rat brain, as determined by immunoblot analysis using SALM4-specific antibodies and different tissue lysates (Fig. 1b,c and Supplementary Fig. 1a–c). SALM4 protein expression gradually increased during postnatal brain development (Fig. 1d and Supplementary Fig. 1d). SALM4 proteins were detected in synaptic brain fractions, including crude synaptosomes, synaptic membranes and PSD fractions (Fig. 1e,f and Supplementary Fig. 1e,f), consistent with the previously reported ultrastructural localization of SALM4 proteins around cell junctions, including neuronal synapses^20^.

### Generation and basic characterization of *Salm4* ^−/−^ mice

**Figure 2:**
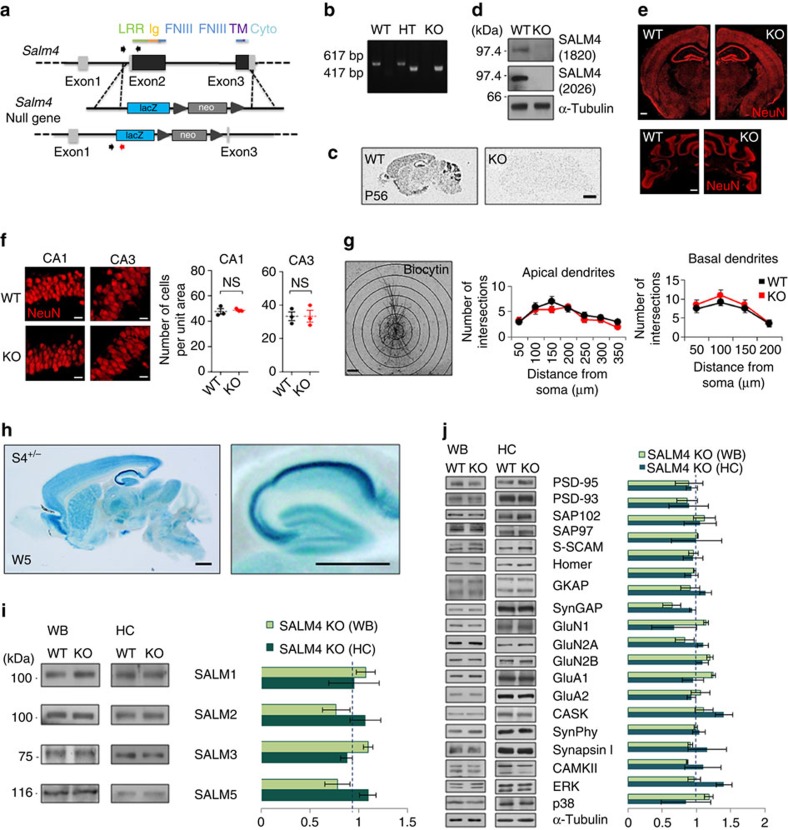
Generation and basic characterization of *Salm4*^−/−^ mice. (**a**) Strategy used to generate *Salm4*^−/−^ mice. LacZ, β-galactosidase gene. Arrows flanking the neomycin gene indicate loxP sites. Arrows at the start of Exon 2 indicate primer sites for PCR genotyping. Note that the lacZ and neo are two separate markers. (**b**) PCR genotyping of *Salm4*^−/−^ (KO), *Salm4*^+/−^ (HT) and WT mice. (**c**) Lack of *in situ* hybridization signals in the *Salm4*^−/−^ brain (P56). (**d**) *Salm4*^−/−^ brain lacks detectable SALM4 proteins, as shown by two different SALM4 antibodies (1820 and 2026 guinea pig). (**e**) Normal gross morphology of *Salm4*^−/−^ brain regions (left, forebrain coronal section; right, cerebellar coronal section). Scale bar, 500 μm. (**f**) Normal numbers of neurons in hippocampal CA1 and CA3 regions. Scale bar, 20 μm. (**g**) Normal morphology of the apical and basal dendrites of *Salm4*^−/−^ hippocampal CA1 pyramidal neurons. Neuronal infusion of biocytin was followed by the analysis of apical (middle) and basal (right) dendritic complexity by Sholl analysis. Scale bar, 50 μm. (**h**) Distribution patterns of SALM4 proteins in the mouse brain, as revealed by X-gal staining of sagittal *Salm4*^+/−^ brain slices at 5 weeks. Scale bars, 1 mm. (**i**) SALM4 deletion does not induce compensatory changes in other SALMs, as determined by immunoblot analysis of *Salm4*^−/−^ whole brain (WB) and hippocampal lysates (3 weeks). *n*=3 mice, ns, Student's *t*-test. (**j**) SALM4 deletion does not cause significant changes in the levels of synaptic proteins (scaffolds, receptors and signalling molecules). *Salm4*^−/−^ WB and hippocampal (HC) lysates (3 weeks) were immunoblotted by the indicated antibodies. *n*=3 mice, ns, Student's *t*-test.

To explore the *in vivo* functions of SALM4, we generated *Salm4*^−/−^ mice (*Salm4*^−/−^ knockout (KO) mice) lacking *Salm4* exons 2 and 3, which encode the full-length SALM4 protein (Fig. 2a,b). SALM4 mRNAs were undetectable in the *Salm4*^−/−^ brain, as determined by *in situ* hybridization (Fig. 2c). SALM4 proteins were also undetectable, as determined by two different SALM4 antibodies (Fig. 2d and Supplementary Fig. 1g). The *Salm4*^−/−^ brain had normal gross morphology and neuronal density in the hippocampus (Fig. 2e,f).

Because SALM4 is known to regulate neurite outgrowth and branching in cultured hippocampal neurons^16^,^17^, we examined the morphology of *Salm4*^−/−^ CA1 pyramidal neurons in the hippocampus using biocytin dye injection and Sholl analysis. We found that *Salm4*^−/−^ neurons had normal dendritic complexities in both apical and basal dendrites (Fig. 2g).

Assessment of SALM4 protein expression by X-gal staining of *Salm4*^+/−^ brain slices (5-week-old mice) revealed that SALM4 could be observed in various regions, including the neocortex, striatum, hippocampus and cerebellum (Fig. 2h). Notably, SALM4 X-gal signals in the hippocampus were stronger in the CA1 and CA3 regions than in the dentate gyrus. This contrasts with the similar levels of SALM4 mRNAs in the CA1/3 and dentate gyrus regions (Figs 1a and 2c), indicative of different expression levels of SALM4 mRNAs and proteins in different brain regions, similar to those observed in the cerebellum. SALM4 deletion had no effect on the levels of other SALMs or synaptic proteins, either in the whole brain or the hippocampus (Fig. 2i,j and Supplementary Fig. 1h).

### *Salm4* ^−/−^ mice display increased synapse numbers

**Figure 3:**
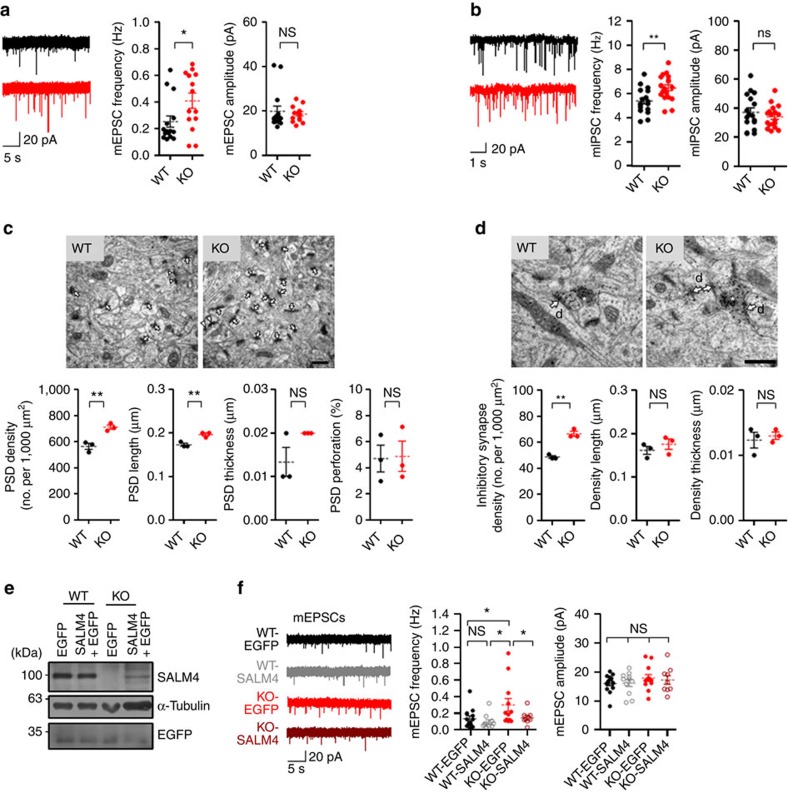
Increased excitatory and inhibitory synapse numbers in the *Salm4*^−/−^ hippocampal CA1 region. (**a**,**b**) The frequency, but not amplitude, of mEPSCs (**a**) and mIPSCs (**b**) is increased in *Salm4*^−/−^ hippocampal CA1 pyramidal neurons (P15–20). *n*=15 cells (three mice) for WT and 14 (three mice) for KO for mEPSCs; *n*=17 (three mice) for WT and 18 (three mice) for KO for mIPSCs, *P<0.05, **P<0.01, ns, not significant, Student's *t*-test. (**c**) PSD density and length, but not thickness or perforation, are increased in the *Salm4*^−/−^ hippocampal CA1 region (P14–21), as determined by EM analysis. Normal and perforated PSDs are indicated by arrows and arrowheads, respectively. Scale bar, 500 nm. *n*=3 mice for WT and KO, **P<0.01, ns, not significant, Student's *t*-test. (**d**) The density, but not the length or thickness, of symmetric, electron-dense synaptic structures is increased in the *Salm4*^−/−^ hippocampal CA1 region (P14–21). GABAergic terminals, indicated by asterisks, are visualized by EM immunogold staining for GABA. Postsynaptic dendrites (*d*) are indicated. Scale bar, 500 nm. *n*=3 mice for WT and KO, **P<0.01, ns, not significant, Student's *t*-test. (**e**,**f**) SALM4 re-expression rescues the increased mEPSC frequency in *Salm4*^−/−^ CA1 neurons. *Salm4*^−/−^ or WT neurons were infected with HSV carrying SALM4+EGFP, or EGFP alone (P15–18), followed by mEPSC measurements (**f**). SALM4/EGFP protein expression was confirmed by immunoblotting (**e**). Note that SALM4 protein levels in the two WT neurons (lanes 1 and 2) are not significantly different (Supplementary Fig. 2b) because a small fraction of total neurons are infected, although this does not affect mEPSC measurements using EGFP-positive neurons. *n*=14 cells (five mice) for WT-EGFP, 11 (five mice) for WT-SALM4, 12 (six mice) for KO-EGFP and 9 (three mice) for KO-SALM4, *P<0.05, ns, not significant, analysis of variance (ANOVA).

Electron microscopic (EM) studies previously detected SALM4 signals in axons, dendrites and synapses^20^, suggesting that SALM4 may regulate synapse development and function. We thus measured spontaneous excitatory synaptic transmission in hippocampal CA1 pyramidal neurons. We found that the frequency (but not the amplitude) of miniature excitatory postsynaptic currents (mEPSCs) was significantly increased in *Salm4*^−/−^ neurons (Fig. 3a). A similar increase was also observed in the frequency (but not amplitude) of miniature inhibitory postsynaptic currents (mIPSCs; Fig. 3b). We could not detect any changes in the input–output relationship of evoked excitatory synaptic transmission and the paired pulse facilitation at *Salm4*^−/−^ Schaffer collateral-CA1 pyramidal synapses (Supplementary Fig. 2), which is not in line with the increased frequency of mEPSCs (Fig. 3a). It is possible that mEPSCs in CA1 pyramidal neurons represent synaptic inputs other than those from the Schaffer collateral pathway (that is, the temporoammonic pathway), or that evoked EPSCs are less sensitive in detecting the changes in excitatory synapse number.

EM analysis of the *Salm4*^−/−^ hippocampus (CA1 stratum radiatum) revealed significant increases in the density and length of the PSD apposed to presynaptic vesicles, whereas there was no change in the thickness or perforation (a measure of maturation) of the PSD (Fig. 3c). In addition, the density of symmetrically electron-dense regions apposed to GABA (γ-aminobutyric acid)-positive inhibitory nerve terminals was increased, whereas there was no change in their length or thickness (Fig. 3d). These results suggest that SALM4 deletion increases the number of excitatory and inhibitory synapses in the hippocampus.

### SALM4 re-expression rescues excitatory synapse numbers

To determine whether the increased synapse number observed in *Salm4*^−/−^ neurons was caused by SALM4 deficiency, we re-expressed SALM4 in *Salm4*^−/−^ CA1 pyramidal neurons by herpes simplex virus (HSV) infection on postnatal days (P) 15–18 (Supplementary Fig. 3a). We found that SALM4 re-expression, confirmed by immunoblot analysis (Fig. 3e and Supplementary Figs 1i and 3b), rescued the increased mEPSC frequency in *Salm4*^−/−^neurons to a normal range without affecting the amplitude (Fig. 3f). In contrast, SALM4 overexpression in wild-type (WT) neurons had no effect on the frequency or amplitude of mEPSCs, suggesting that endogenous levels of SALM4 proteins are sufficient to have the full negative effect on mEPSC frequency.

In contrast, SALM4 expression in *Salm4*^−/−^ CA1 pyramidal neurons had no effect on the frequency or amplitude of mIPSCs (Supplementary Fig. 3c). Moreover, *Salm4*^−/−^ neurons expressing enhanced green fluorescence protein (EGFP) showed mIPSC frequencies similar to those of WT neurons expressing EGFP, which is in contrast to the increased mIPSC frequency observed in uninfected *Salm4*^−/−^ neurons. This might be attributable to the viral infection, which may have adverse effects on inhibitory (but not excitatory) synaptic transmission under our experimental conditions.

### SALM4 *cis*-interacts with SALMs 2/3/5

**Figure 4:**
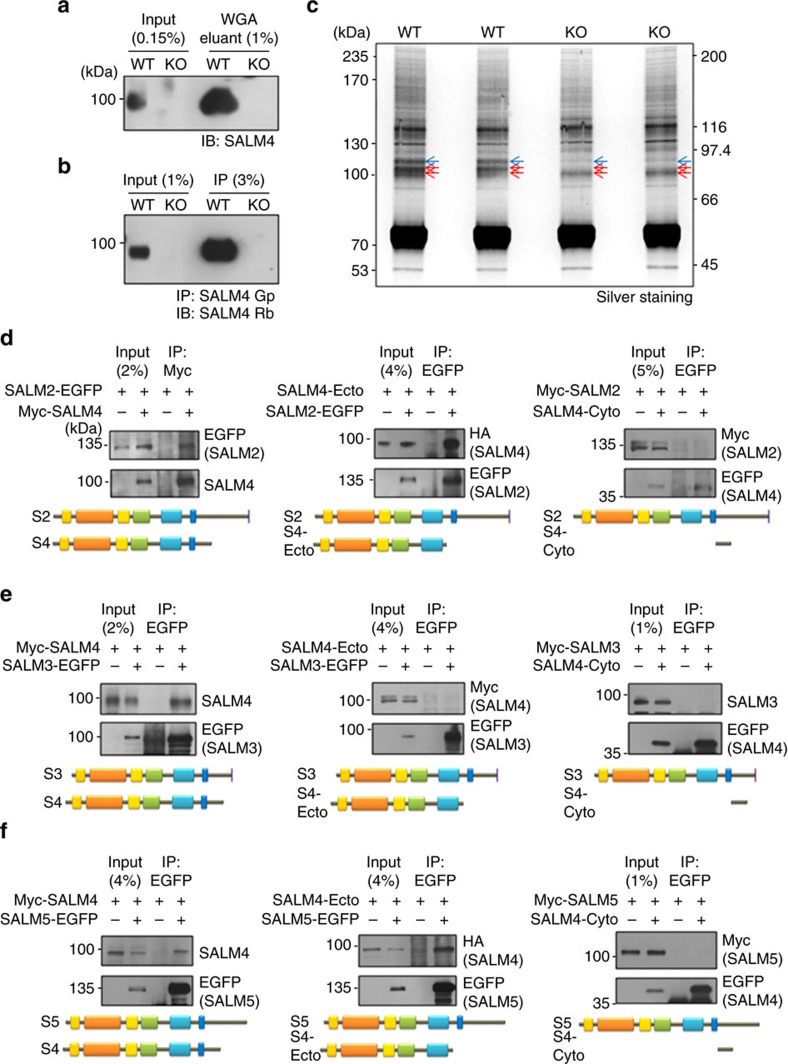
SALM4 *cis*-interacts with SALMs 2/3/5. (**a**) Enrichment of mature membrane proteins from forebrain crude synaptosomes (P21) using a WGA column. (**b**,**c**) The WGA eluants were immunoprecipitated (IP) with SALM4 antibodies (2026) and then subjected to preparative SDS–PAGE (**c**). A small aliquot of each precipitate was immunoblotted (IB) for SALM4 to confirm self-immunoprecipitation (**b**). The three bands (arrows) in **c** contained peptides of SALM4 (all three) and SALM2 (top blue). (**d**–**f**) SALM4 co-precipitates with SALM2/3/5 in heterologous cells. HEK293T cell lysates doubly transfected with SALM4 (Myc full-length, HA/Myc ectodomain or EGFP cytoplasmic domain)+EGFP-SALM2/3/5 (EGFP/Myc full-length) were IP and immunoblotted with the indicated antibodies. HA/Myc-SALM4 ectodomain represents SALM4 in the pDisplay vector tagged by both HA and Myc.

To better understand the molecular mechanisms underlying the SALM4-dependent negative regulation of neuronal synapses, we performed an unbiased proteomic screen of SALM4-associated proteins in WT and *Salm4*^−/−^ (control) mouse forebrains. We first enriched mature membrane proteins, including SALM4, using a wheat germ agglutinin (WGA) column (Fig. 4a). When these preparations were immunoprecipitated with SALM4 antibodies and subjected to proteomic analyses, we identified SALM2 as a major protein associated with SALM4 in WT brains but absent in *Salm4*^−/−^ immunoprecipitates (of the total 20 peptides, 12 were SALM4 and 8 were SALM2; Fig. 4b,c).

This identification of an *in vivo* association between SALM4 and SALM2 contrasts with the previous reports that SALM1–3, but not SALM4 or -5, form complexes with one another in the rat brain^20^. This discrepancy might reflect that our antibodies are somehow more efficient in pulling down SALM4 proteins in complex with SALM2. Indeed, a previous study reported that SALM1–3 exhibited antibody-dependent differential co-immunoprecipitation, wherein SALM1 immunoprecipitates contained almost undetectable amounts of SALM2 and SALM3, but SALM2 and SALM3 immunoprecipitates contained significant amounts of SALM1 (ref. 20).

To further characterize the interaction between SALM4 and SALM2, we performed co-immunoprecipitation experiments in heterologous cells. We found that SALM4 formed a complex with SALM2 (Fig. 4d and Supplementary Fig. 4a). In addition, the extracellular (ecto) domain of SALM4 (but not the cytoplasmic domain; SALM4-Ecto) could associate with SALM2, suggesting that the ecto domains of SALM4 and SALM2 are involved.

SALM2 forms a complex with SALM3 *in vitro* and *in vivo* and with SALM5 *in vitro*^20^. In addition, SALM3 and SALM5 (but not the other SALMs) have synaptogenic activities^18^. We therefore hypothesized that SALM4 might suppress neuronal synapses through a *cis*-interaction with SALM3 and/or SALM5. To examine this hypothesis, we first tested whether SALM4 could associate with SALM3 and SALM5, in addition to SALM2.

We found that SALM4 forms a complex with SALM3 in heterologous cells, but neither the ecto nor cytoplasmic domain of SALM4 was sufficient to mediate the interaction (Fig. 4e and Supplementary Fig. 4b), dissimilar to the domain requirement of the SALM4–SALM2 interaction. However, both full-length and ectodomain of SALM4 could associate with SALM5 (Fig. 4f and Supplementary Fig. 4b), similar to the SALM4–SALM2 interaction.

**Figure 5:**
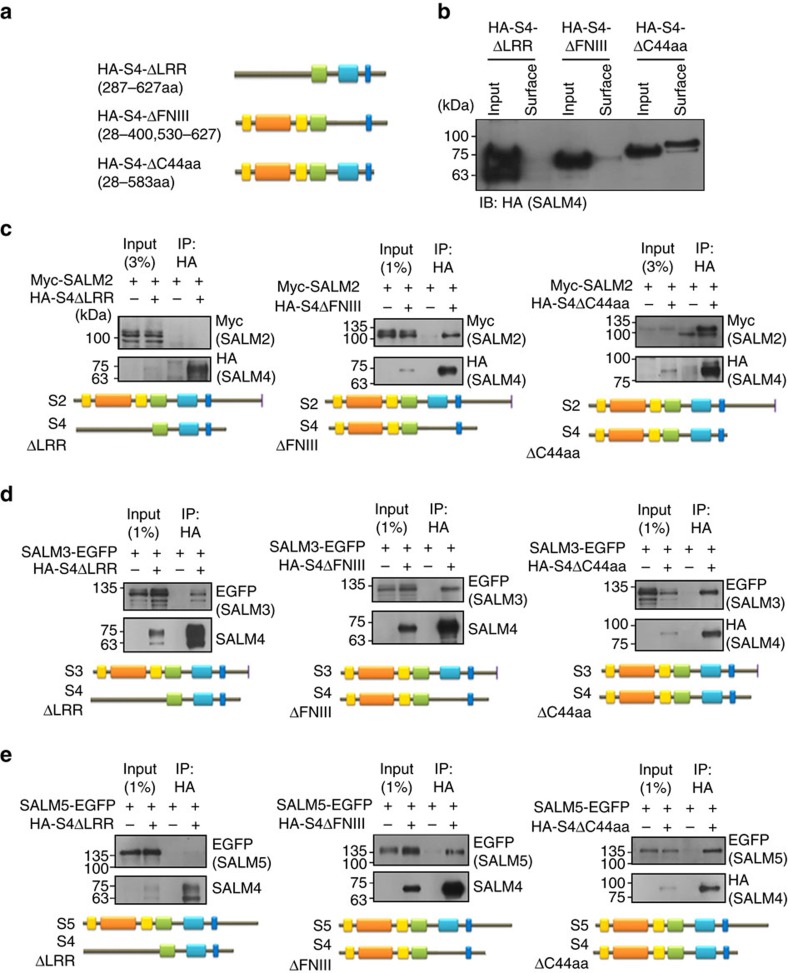
Domains of SALM4 involved in the interaction with SALM2, SALM3 and SALM5. (**a**) Deletion variants of HA-SALM4 (HA-S4). FL, full length. (**b**) Total and surface expression levels of SALM4 deletion variants. HEK293T cells expressing HA-SALM4 deletion variants were biotinylated, precipitated and immunoblotted. (**c**–**e**) Deletion variants of HA-SALM4 differently interact with SALM2/3/5 in heterologous cells. HEK293T cell lysates doubly transfected with HA-SALM4 (WT and deletion variants)+SALM2/3/5 (Myc-SALM2, SALM3-EGFP and SALM5-EGFP) were immunoprecipitated and immunoblotted with the indicated antibodies.

In order to further narrow down the domains of SALM4 involved in the interactions with SALM2, SALM3 and SALM5, we generated additional deletion variants of SALM4 that lacks the LRR domain (SALM4-ΔLRR), the FNIII domain (SALM4-ΔFNIII) and a large portion of the cytoplasmic region (SALM4-ΔC44aa), which displayed normal total expression levels but markedly reduced surface expression levels in SALM4-ΔLRR and SALM4-ΔFNIII but not in SALM4-ΔC44aa (Fig. 5a,b and Supplementary Fig. 4c). Assuming that non-surface SALM4 proteins may still interact other SALMs in cytoplasmic compartments, we performed co-immunoprecipitation experiments and found that SALM4-ΔLRR interacted with SALM3 but not with SALM2 or SALM5 (Fig. 5c–e and Supplementary Fig. 4d). In addition, SALM4-ΔFNIII and SALM4-ΔC44aa interacted with all three SALMs (SALM2, SALM3 and SALM5; Fig. 5c–e). These results collectively suggest that the LRR domain of SALM4 is important for the interaction with SALM2 and SALM5, while the transmembrane domain of SALM4 is important for the interaction with SALM3.

### SALM4 inhibits synapse-promoting effects of SALM2/3/5

**Figure 6:**
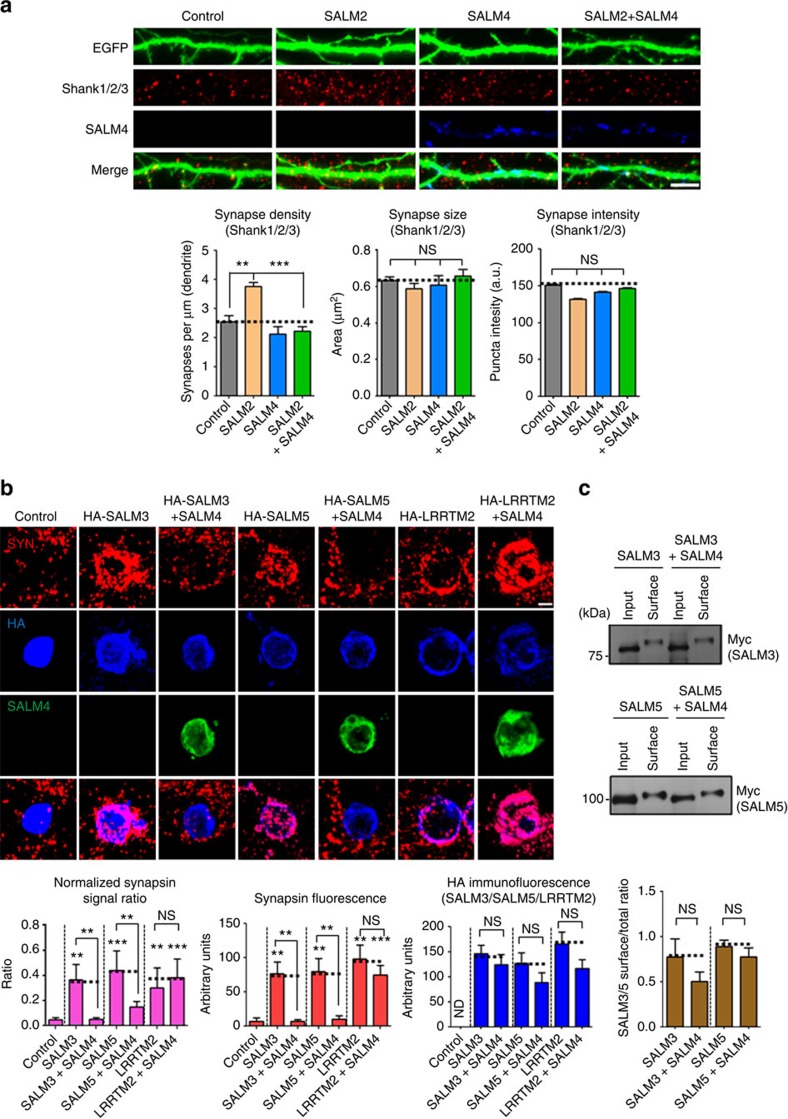
SALM4 inhibits SALM2-dependent excitatory synapse facilitation and SALM3/5-dependent presynaptic differentiation. (**a**) SALM4 inhibits SALM2-dependent promotion of excitatory synapse number. (Top panels) Representative images of cultured hippocampal neurons transfected with control (EGFP alone), SALM2 and EGFP (SALM2), SALM4 and EGFP (SALM4) or co-transfected with SALM2 and SALM4 (SALM2+SALM4) at DIV10. Neurons were analysed at DIV14 by triple immunofluorescence for EGFP (green), Shank (an excitatory postsynaptic marker protein; red) and SALM4 (blue). Scale bar, 10 μm, applies to all images. (Bottom panels) Bar graphs summarizing the effects of SALM4 overexpression on SALM2-induced postsynaptic development, quantified using Shank immunoreactivity (pan-Shank). *n*=15 for control (EGFP alone), SALM2, SALM4 and SALM2+SALM4, **P<0.01, ***P<0.001, ns, not significant, ANOVA with Tukey's test. (**b**) SALM4 suppresses SALM3/5-dependent presynaptic differentiation. (Top panels) Representative images of cocultures. Hippocampal neurons were cocultured for 2 days (DIV 10–12) with HEK293T cells expressing EGFP alone (control), SALM3/5, SALM3/5+SALM4, LRRTM2 or LRRTM2+SALM4, and stained for EGFP/HA (blue), SALM4 (green) and synapsin I (red). Scale bar, 10 μm. (Bottom panels) Quantification of heterologous synapse-forming activities of SALM3/5 and LRRTM2, by measuring the ratio of synapsin I to surface HA immunofluorescence (absolute red/synapsin and blue/HA fluorescence values are also indicated). Note that SALM4 coexpression does not affect the surface expression of SALM3, SALM5 or LRRTM2, as indicated by fluorescence intensity of HA signals. *n*=12 for control, 11 for SALM3, 10 for SALM3+SALM4, 10 for SALM5, 10 for SALM5+SALM4, 12 for LRRTM2 and 11 for LRRTM2+SALM4, **P<0.01, ***P<0.001, ns, not significant, ANOVA with Tukey's test. (**c**) SALM4 does not affect surface levels of SALM3 or SALM5, as determined by surface biotinylation assays. HEK293T cells transfected with SALM3/5 alone, or SALM3/5+SALM4, were biotin-labelled, followed by avidin precipitation and immunoblotting. Input and surface, 1% and 8%, respectively. *n*=3 for SALM3 and SALM3+SALM4, ns, not significant, Student's *t*-test.

Because SALM4 associates with SALM2, SALM3 and SALM5, we tested whether SALM4 regulates the functional properties of SALM2/3/5. SALM2 has been shown to facilitate excitatory synapse differentiation, as shown by that cultured hippocampal neurons overexpressing SALM2 show increased excitatory synapse number^13^. When we coexpressed SALM2 and SALM4 in cultured hippocampal neurons, SALM4 reversed the SALM2-dependent increase in excitatory synapse number, as indicated by the density of Shank1/2/3 (Fig. 6a), an excitatory postsynaptic marker protein. In contrast, the size and intensity of Shank clusters were not changed by either the overexpression of SALM2 or SALM2+SALM4. This suggests that SALM4 suppresses excitatory synapse-promoting effect of SALM2.

SALM3 and SALM5 have been shown to promote presynaptic differentiation in contacting axons^18^. We thus tested whether SALM4 could inhibit SALM3- or SALM5-dependent presynaptic differentiation, using neuron–fibroblast-mixed culture assays^21^. We found that coexpression of SALM4 with SALM3 in heterologous cells markedly suppressed SALM3-dependent synapsin I clustering in contacting axons of cocultured hippocampal neurons (Fig. 6b). SALM4 had similar effects on SALM5-dependent synapsin I clustering. In control experiments, SALM4 coexpression had no effect on synapsin I clustering induced by LRRTM2, an LRR-containing adhesion molecule with synaptogenic activity^22^,^23^. SALM4 expression did not change the surface levels of coexpressed SALM3, SALM5 or LRRTM2, although there were decreasing tendencies, as determined by surface immunofluorescence staining (Fig. 6b) and surface biotinylation assays (Fig. 6c and Supplementary Fig. 5a).

### SALM4 inhibits the interaction between SALM3/5 and LAR

**Figure 7:**
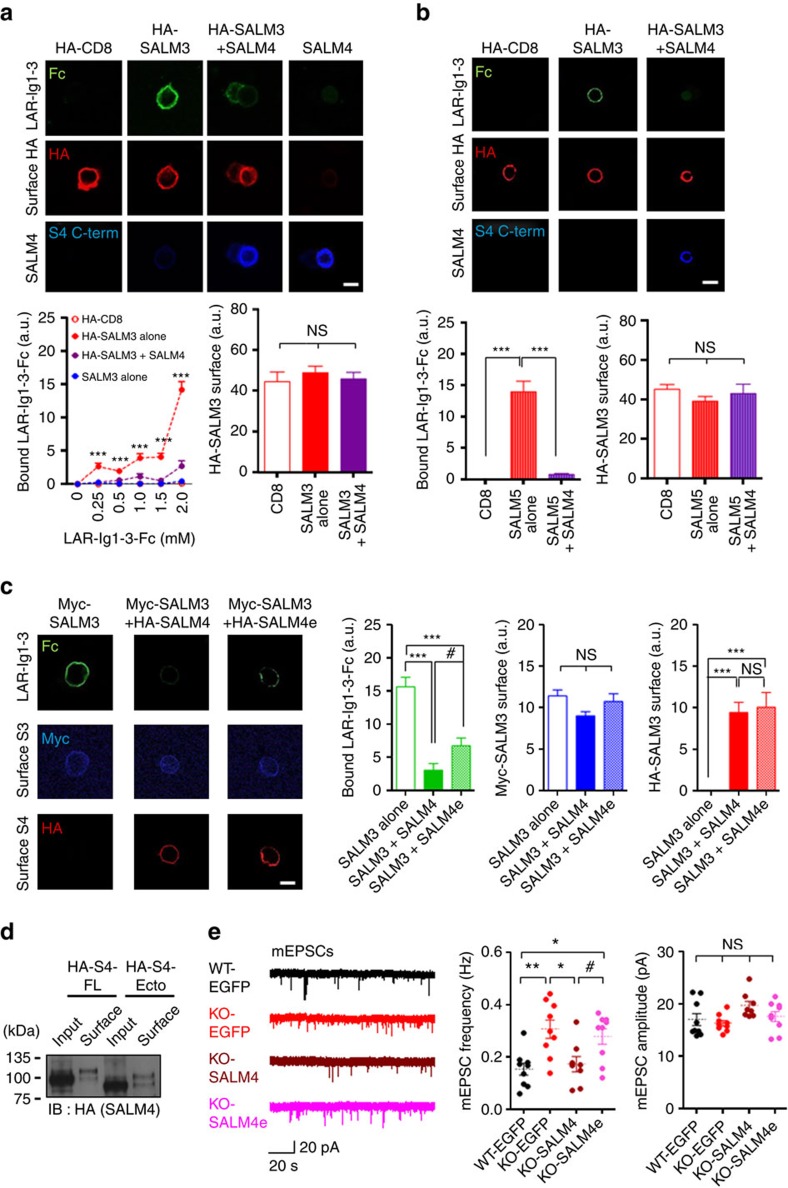
SALM4 inhibits the interaction between SALM3/5 and LAR. (**a**) Soluble LAR binding to SALM3 on heterologous cells is reduced by coexpression of SALM4. HEK293T cells transfected with HA-CD8 (control), SALM3 alone or SALM3+SALM4 were incubated with LAR-Ig1-3-Fc (Ig1-3 domains of LAR fused to human Fc) at increasing concentrations, followed by staining for Fc (bound LAR-Ecto-Fc), HA (CD8 or SALM3; red) and SALM4 (blue). For quantification, signals for bound LAR-Ig1-3-Fc were normalized by surface HA-SALM3 signals. Surface expression of HA-SALM3 is not affected by SALM4 coexpression (bottom right). *n*=18 cells for CD8, 23 for S3, 20 for S3+S4 and 19 for S4 (0.25 mM); 8, 13, 18 and 18 (0.5 mM); 17, 27, 26 and 22 (1 mM); 13, 27, 19 and 19 (1.5 mM); 23, 27, 32 and 15 (2 mM). ***P<0.001 (relative to SALM3+SALM4 or SALM4 alone), ns, not significant, ANOVA. Scale bar, 20 μm. (**b**) LAR binding to SALM5 is reduced by coexpression of SALM4. Experiments were performed as in **a**, except using SALM5 rather than SALM3 and a single concentration of LAR-Ig1-3-Fc (2 mM) for simplicity. *n*=53 for CD8, 46 for S5 and 41 for S5+S4, ***P<0.001, ns, not significant, ANOVA. Scale bar, 20 μm. (**c**,**d**) SALM4-Ecto (*SALM4e*) that lacks SALM3 binding, replacing full-length SALM4, shows partially reverses SALM4-dependent suppression of LAR binding to SALM3. SALM4 or SALM4e does not affect surface expression of SALM3, as shown by biotinylation of surface proteins (**d**). *n*=34 for S3, 28 for S3+S4 and 30 for S3+S4e, ***P<0.001, ns, not significant, ANOVA and ^#^*P*<0.05, Student's *t*-test. Scale bar, 20 μm. (**e**) SALM4-Ecto, replacing full-length SALM4, substantially reverses SALM4-dependent rescue of the increased mEPSC frequency in *Salm4*^−/−^ CA1 neurons. WT neurons were infected with HSV carrying EGFP, and *Salm4*^−/−^ neurons were infected with HSV carrying EGFP, EGFP+HA-SALM4 and EGFP+HA-SALM4-Ecto (P15–18), followed by mEPSC measurements. *n*=9 cells (three mice) for WT-EGFP, 9 (three mice) for KO-EGFP, 8 (three mice) for KO-SALM4 and 9 (three mice) for KO-SALM4e, *P<0.05, **P<0.01, ns, not significant, ANOVA and ^#^*P*<0.05, Student's *t*-test.

Because SALM4 suppresses SALM3- and SALM5-dependent presynaptic differentiation, we next tested whether SALM4 could inhibit the interaction between SALM3 and its known presynaptic ligand, LAR^19^, or that between SALM5 and LAR^24^. To this end, we tested whether the binding of LAR (a soluble form) to SALM3 displayed on heterologous cell surfaces could be inhibited by SALM4 coexpressed in the same cell. Indeed, the interaction of SALM3 with increasing concentrations of soluble LAR (extracellular Ig1-3 domains; LAR-Ig1-3-Fc) was significantly reduced by SALM4 coexpression (Fig. 7a). Similarly, LAR binding to SALM5 was significantly inhibited by SALM4 coexpression (Fig. 7b).

In control experiments, we used a mutant SALM4 that lacks SALM3 binding (SALM4-Ecto; see Fig. 4e) to see whether it fails to inhibit LAR binding to SALM3. We found that SALM4-Ecto partially reversed full-length SALM4-dependent suppression of LAR binding to SALM3 (Fig. 7c). SALM4-Ecto did not affect the surface expression of SALM3 (Fig. 7d and Supplementary Fig. 5b).

In another set of experiments measuring mEPSCs in HSV-infected hippocampal neurons, SALM4-Ecto substantially reversed full-length SALM4-dependent normalization of the increased mEPSC frequency in *Salm4*^−/−^ CA1 pyramidal neurons (Fig. 7e, Supplementary Fig. 3d–f; Supplementary Fig. 5c,d). These results collectively suggest that SALM4 suppresses SALM3- and SALM5-dependent presynaptic differentiation through *cis*-interactions.

### Double *Salm3*-*Salm4* KO normalizes excitatory synapse numbers

**Figure 8:**
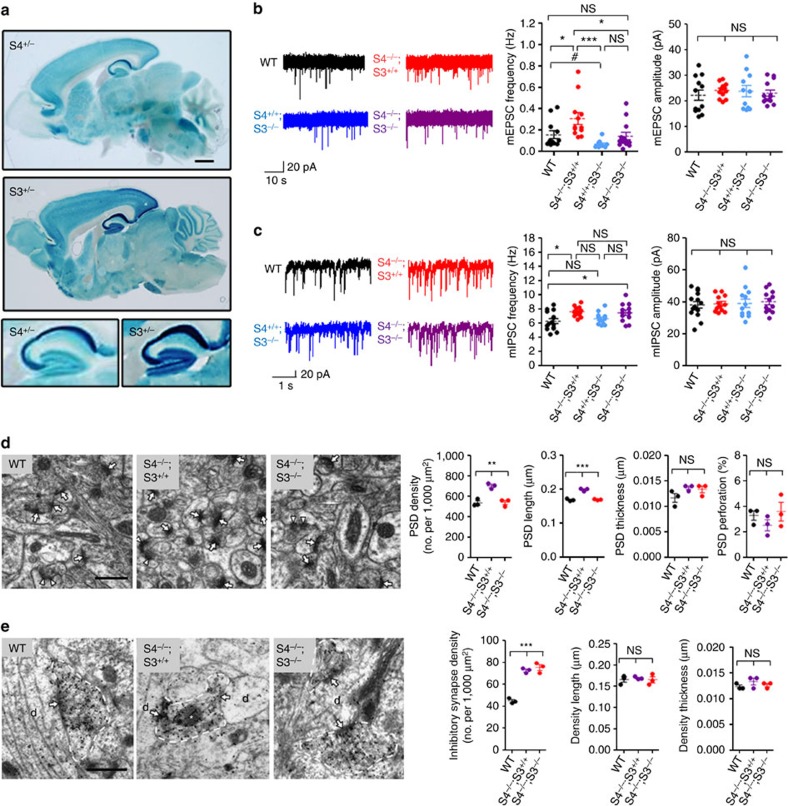
Double KO of SALM3 and SALM4 normalizes excitatory synapse numbers in both *Salm4*^−/−^ and *Salm3*^−/−^ neurons. (**a**) Comparison of SALM3 and SALM4 protein expression patterns in sagittal brain sections by X-gal staining of *Salm3*^+/−^ and *Salm4*^+/−^ single KO slices (5 weeks). Note that the hippocampal CA3 and CA1 signals are stronger than those in the dentate gyrus. Scale bar, 1 mm. (**b**) *Salm3*^−/−^; *Salm4*^−/−^ CA1 pyramidal neurons display mEPSC frequencies comparable to those of WT neurons (P18–21), indicative of normalization in both in *Salm4*^−/−^ and *Salm3*^−/−^ neurons, although such effects are weaker in *Salm3*^−/−^ neurons. Note that the double KO has no effect on mEPSC amplitudes. *n*=12 cells (four mice) for WT, 11 (three mice) for *Salm3*^−/−^, 12 (three mice) for *Salm4*^−/−^ and 13 (four mice) for *Salm3*^−/−^; *Salm4*^−/−^, *P<0.05, **P<0.01, ***P<0.001, ns, not significant, ANOVA with Bonferroni' multiple comparison test; ^#^*P*<0.05, Student's *t*-test. (**c**) The double KO does not rescue the increased mIPSC frequency in *Salm4*^−/−^ CA1 pyramidal neurons. *n*=15 (four mice) for WT, 13 (three mice) for *Salm3*^−/−^, 12 (three mice) for *Salm4*^−/−^ and 12 (four mice) for *Salm3*^−/−^; *Salm4*^−/−^, *P<0.05, ns, not significant, ANOVA with Bonferroni' multiple comparison test. (**d**) The increased PSD density in the *Salm4*^−/−^ hippocampus is normalized by the double KO, as shown by the EM analysis of the hippocampal CA1 stratum radiatum region (P20). Normal and perforated PSDs are indicated by arrows and arrowheads, respectively. Scale bar, 500 nm. *n*=3 mice for WT, *Salm4*^−/−^, and *Salm3*^−/−^;*Salm4*^−/−^, *P<0.05, **P<0.01, ns, not significant (*P*=0.1551 for PSD thickness and 0.3996 for perforated spines), ANOVA. (**e**) The increased density of symmetric, electron-dense synaptic structures in the *Salm4*^−/−^ hippocampus is not normalized by the double KO, as shown by the EM analysis of the hippocampal CA1 stratum radiatum region (P20). GABAergic terminals (asterisks) are indicated by EM immunogold staining for GABA. Postsynaptic dendrites (d) are also indicated. Scale bar, 500 nm. *n*=3 mice for WT, *Salm4*^−/−^, and *Salm3*^−/−^; *Salm4*^−/−^, ***P<0.001, ns, not significant, ANOVA.

The *cis*-regulation of SALM3/5 by SALM4 requires their expression in the same cells. In line with this, X-gal staining revealed that the expression patterns of SALM3 and SALM4 proteins significantly overlapped in mouse brain regions, including the neocortex, hippocampus and striatum (Fig. 8a and Supplementary Fig. 6a), and also in hippocampal CA3 and CA1 regions. The potential overlap between SALM4 and SALM5 could not be tested because *Salm5*^−/−^ mice that allow X-gal staining were unavailable; however, we note that the known mRNA distribution pattern of SALM5 substantially overlap with those of SALM3 and SALM4 (refs 13, 14).

Next, we hypothesized that if SALM4-dependent suppression of excitatory synapse number involves the inhibition of SALM3/5, removal of SALM3/5 might rescue the increased excitatory synapse number observed in *Salm4*^−/−^ neurons. To this end, we crossed *Salm3*^−/−^ mice, which we recently reported to exhibit reduced excitatory synapse numbers in the hippocampus^19^, with *Salm4*^−/−^ mice to obtain double KO mice (Supplementary Figs 6b and 5e). *Salm3*^−/−^;*Salm4*^−/−^ mice and *Salm3*^−/−^ mice (but not *Salm4*^−/−^ mice) displayed slightly lower body weights than controls (Supplementary Fig. 6c).

We found that the increased mEPSC frequency observed in *Salm4*^−/−^ CA1 pyramidal neurons was normalized to near-WT levels in *Salm3*^−/−^; *Salm4*^−/−^ CA1 pyramidal neurons (Fig. 8b). This result also indicates that the reduced mEPSC frequency in *Salm3*^−/−^ neurons was normalized in the double KO mice, although the extent of normalization was weaker than that for *Salm4*^−/−^ neurons. The double KO had no effect on the amplitude of mEPSCs.

When mIPSCs were compared, we found that the double KO did not rescue the increased mIPSC frequency seen in *Salm4*^−/−^ mice (Fig. 8c), nor did it affect the mIPSC frequency in *Salm3*^−/−^ mice. Moreover, the observed mIPSC amplitudes were comparable across the four genotypes. Together, these results indicate that the double KO of SALM3 and SALM4 rescues the increased frequency of mEPSCs (but not mIPSCs) in *Salm4*^−/−^ mice.

We next performed EM analysis to see whether the double KO normalizes the increased excitatory and inhibitory synapse densities in *Salm4*^−/−^ mice. We found that the *Salm3*^−/−^;*Salm4*^−/−^ hippocampus (CA1 stratum radiatum) shows the density of the PSD significantly smaller than that of *Salm4*^−/−^ hippocampus but comparable to that of WT hippocampus (Fig. 8d). In contrast, the increased inhibitory synapse density in *Salm4*^−/−^ hippocampus was not normalized by the double KO (Fig. 8e). These results further suggest that SALM3 mediates the SALM4-dependent inhibition of excitatory synapse number.

## Discussion

In the present study, we report that *Salm4*^−/−^ mice display increased excitatory and inhibitory synapse numbers in the hippocampus, and that SALM4 *cis*-interacts with SALM3, inhibits the SALM3–LAR interaction and suppresses SALM3-dependent presynaptic differentiation.

Our data indicate that a SALM4 mutant that lacks SALM3 binding (SALM4-Ecto), unlike full-length SALM4, loses the ability to inhibit LAR binding to SALM3 in HEK293T cells, and to rescue the increased mEPSC frequency in CA1 pyramidal cells (Fig. 7d–f). However, the extent of these reversals are partial, especially in the LAR-binding experiments *in vitro*. One possibility is that SALM4-Ecto, although it no longer associates with SALM3 in HEK293T cells under co-immunoprecipitation conditions (Fig. 4e), may still weakly associate with, or get sufficiently close to, SALM3 on the surface membrane through its intact extracellular domain and inhibit LAR binding to SALM3; the fact that SALM4-ΔLRR binds to SALM3 does not exclude the possibility that the LRR domain of SALM4 may still weakly bind to SALM3. This nonspecific inhibition, if present, might be stronger when SALM4-Ecto is highly expressed in HEK293T cells, whereas it is weaker when SALM4-Ecto is weakly expressed in neurons, or SALM4-Ecto cannot easily have an access to endogenous SALM3 at excitatory synaptic sites.

Our hypothesis (SALM3 inhibition by SALM4) is genetically supported by our observation that double KO of SALM3 and SALM4 normalizes mEPSC frequency and excitatory synapse number phenotypes. This suggests that SALM3, but not SALM5, may act as a major downstream effector of SALM4 for the inhibition of excitatory synapse number in the hippocampus. In line with this possibility, SALM3 and SALM4 show substantial overlaps in their protein distribution patterns in the brain (Fig. 8a). Moreover, a previous study showed that the temporal expression patterns of SALM3 and SALM4 mRNAs along the embryonic developmental stages are more similar than those of the other SALMs^14^. These results, however, do not exclude the possibility that SALM5 may closely interact with SALM4 in some other brain regions.

Our data, however, provide relatively few clues as to how SALM4 negatively regulates inhibitory synapses. The increased inhibitory synapse number in *Salm4*^−/−^ mice might merely represent a compensatory change induced by the increase in excitatory synapse number. Alternatively, it could be a more direct effect involving SALM4. In line with this, a previous study showed that SALM3 and SALM5, which associate with SALM4, can induce both excitatory and inhibitory presynaptic differentiation in cocultured neurons^18^. In addition, the LAR family proteins, which bind to SALM3 and SALM5, can induce both excitatory and inhibitory postsynaptic differentiations by interacting with NGL-3, TrkC, IL1RAPL1, IL1RAcP and Slitrks^2^,^6^.

It is likely that ‘postsynaptic' SALM4 *cis*-interacts with SALM3 to suppress excitatory synapses. SALM4 is detected at both pre- and postsynaptic sites, and is thought to mediate homophilic and *trans*-synaptic adhesions^20^. This homophilic adhesion, however, is unlikely to contribute to the SALM4-dependent negative regulation of SALM3 because we herein showed that HSV-mediated re-expression of SALM4 in *Salm4*^−/−^ CA1 pyramidal neurons (postsynaptic side) was sufficient to rescue the increased mEPSC frequency in a cell-autonomous manner.

Our study extends the reported complexity of interactions among the SALM family members. The proteomic data derived from WT and KO (control) brain samples suggest that there is a strong biochemical association between SALM4 and SALM2 *in vivo*. In addition, SALM4 associates with SALM3 and SALM5 in addition to SALM2 in heterologous cells. Given that SALMs 1–3 form a strong co-immunoprecipitable complex in the brain^20^, SALM4, together with SALMs 1–3 and SALM5, may form combinations of protein complexes larger than previously revealed in different brain regions. This complex might be further stabilized by interactions with PSD-95 through SALMs 1–3, and with LAR family proteins through SALMs 3 and 5 (refs 12, 13, 14, 19). Therefore, the SALM4-dependent suppression of SALM2-dependent excitatory synapse development (Fig. 6a) could be mediated indirectly through SALM3. Conversely, the SALM4-dependent suppression of SALM3-dependent presynaptic differentiation could also be mediated or modulated by SALM2, a major binding partner of SALM4 in the brain (Fig. 4b,c).

Inhibition of neuronal synapses via *cis*-interactions of SALM4 is reminiscent of the previously reported *cis*-interaction between neuroligin-1 and neurexin-1β, which suppresses neuroligin-1-dependent presynaptic differentiation in contacting axons^25^. In addition, postsynaptic MDGA1 was shown to *cis*-interact with neuroligin-2 and thereby suppress neuroligin-2-dependent inhibitory presynaptic differentiation^26^,^27^. These results suggest that postsynaptic adhesion molecules may *cis*-interact with one another to regulate their *trans*-interactions. In addition, we recently showed that a *trans*-interaction between postsynaptic NGL-1 and presynaptic netrin-G1 (a glycosylphosphatidylinisotol (GPI)-anchored synaptic adhesion molecule) induces the *cis*-interaction of netrin-G1 with LAR^28^. These results suggest that there may be functional interplays between *cis*- and *trans*-adhesions at the synapse.

SALMs have been implicated in autism spectrum disorders, intellectual disability and schizophrenia^29^,^30^,^31^,^32^,^33^. In addition, LAR family proteins are implicated in autism spectrum disorders, attention deficit/hyperactivity disorder, restless leg syndrome and schizophrenia^2^,^6^. Therefore, the mechanisms revealed in the present study might help us better understand these disorders.

In conclusion, our results suggest that postsynaptic SALM4 negatively regulates excitatory synapse numbers through a *cis*-interaction with SALM3 and the inhibition of both the SALM3–LAR interaction and SALM3-dependent presynaptic differentiation. Our study provides genetic support for the emerging notion that neighbouring adhesion molecules *cis*-interact with one another to regulate neuronal synapses.

## Methods

### Generation of *Salm4* ^−/−^ mice

A mouse ES cell clone (15252A-A12; Lrfn3_AA12), derived from the C57BL/6N strain, was obtained from Velocigene (VG15252). The cassette (ZEN-Ub1) for an allele deletion contained LacZ and neomycin (β-geo) and parts of exons 2 and 3 of the *Salm4/ Lrfn3* gene for homologous recombination. To generate male chimeric mice, cultured ES cells (C57BL/6N) were microinjected into the blastocyst of the C57BL/6J-Tyr<c-2J>(albino B6). Chimeric mice were bred with albino B6 females (C57BL/6J-Tyr<c-2J>) to generate germline-transmitted F0 mice (C57BL/6J-Tyr<c-2J>+C57BL/6N strain). F0 mice were backcrossed to C57BL/6J for two to seven generations. The F2 mice were used for the analysis of brain morphology and synaptic protein levels. Electrophysiology and electron microscopy were performed using F3–7 generations. All mice were bred and maintained according to the KAIST Animal Research Requirements, and all procedures were approved by the Committees of Animal Research at KAIST. Mice were fed *ad libitum* by standard rodent chow and tap water, and housed under 12-h light/dark cycle (lights off at 19:00).

### cDNA constructs

Full-length untagged rat SALM4 (aa 1–626) expression construct was generated by amplifying the insert from a rat brain cDNA library (BD Bioscience Clontech) by PCR and subcloning it into GW1 vector. Haemagglutinin (HA)-tagged full-length mouse SALM3 (aa 28–636) was subcloned into pDisplay vector. Full-length untagged mouse SALM4, Myc-tagged SALMs, EGFP-tagged SALMs and SALM4/5-Ecto constructs have been described previously^18^. Cytoplasmic regions of mouse SALM4 (aa 561–626) were subcloned into pEGFP-C1. The pDisplay-LRRTM2 construct has been described^34^. HA-tagged full-length mouse SALM4 (aa 28–627), SALM4-ΔLRR (aa 287–627), SALM4-ΔFNIII (aa 28–400, 530–627), SALM4-ΔC44aa (aa 28–583) with their own transmembrane domains, cytoplasmic domains and stop codons were subcloned into pDisplay, and SALM4-Ecto (aa 28–530) was subcloned into a modified pDisplay vector lacking the Myc epitope but with an intact HA epitope and transmembrane domain. pIRES2-SALM2-WT-EGFP has been described previously^13^.

### *In situ* hybridization

Mouse brain sections (12 μm thick) at embryonic day (E16 and E18) and postnatal days (P7, P14, P21 and P56) were prepared using a cryostat (Leica CM 1950). Mouse brain sections from WT and *Salm4*^−/−^ mice (P56) were also generated to visualize the lack of SALM4 mRNAs in *Salm4*^−/−^ mice. Hybridization probe specific for mouse SALM4 mRNA was prepared using the following regions: nt 1,429–1,881 of SALM4 (GenBank DQ078787). Antisense riboprobe was generated using 35S-uridine triphosphate (UTP) and the Riboprobe system (Promega).

### Antibodies

SALM4 antibodies (1,820, 1:500 for western blot (WB); 2026, 1:1,000 for WB or purified form 1:200 for immunocytochemistry (ICC); both guinea pigs) were generated by using GST-SALM4 aa 561–626 and 596–626 (last 30 aa; NM_175478) as immunogens, respectively. SALM1 (2,022, 1:500 for WB; guinea pig), SALM2 (2,058, 1:500 for WB; guinea pig) and SALM3 (2,024, 1:500 for WB; guinea pig) antibodies were generated using the last 30 aa of mouse proteins. SALM5 (1,943, 1:500 for WB; guinea pig) antibodies generated using the H_6_-cytoplasmic domain of mouse SALM5 (aa 551–746). EGFP antibodies (1,168, 1:1,000 for WB, not for ICC; rabbit) were generated using the full-length EGFP protein as immunogen. Rabbit polyclonal Shank antibodies, which are specific for all three Shanks (Shank1/2/3, 1:500 for ICC), were generated using H_6_-Shank1-SAM domain (aa 1,852–2,167) as immunogen. The following antibodies have been described previously: SALM3 (1,828, 1:500 for WB; rabbit) and SALM5 (1,907, 1:500 for WB; rabbit)^18^, PSD-95 (1,688, 1:1,000 for WB; guinea pig), SAP102 (1,447, 1:500 for WB; guinea pig), SAP97 (1,443, 1:500 for WB; guinea pig), CaMKII (1,299, purified form,1:2,000 for WB; rabbit)^35^, PSD-93/chapsyn-110 (1,634, 1:1,000 for WB; rabbit), SynGAP (1,682, 1:300 for WB; rabbit), GluR1 (1,193, 1:500 for WB; rabbit), GluR2 (1,195, 1:1,000 for WB; rabbit), CASK (1,640, 1:500 for WB; rabbit)^36^, Homer (1,133, 1:400 for WB; rabbit)^37^, GKAP (1,243,1:1,000 for WB; guinea pig)^38^ and S-SCAM (1,146, 1:1,000 for WB; rabbit)^39^. The following antibodies were purchased from commercial sources: synaptophysin (Santa Cruz, sc-9116, 1:1,000 for WB), GluN1 (BD Biosciences Transduction Laboratories, 556308, 1:1,000 for WB), GluN2A (Invitrogen, A-6473, 1:500 for WB), GluN2B (NeuroMab,N59/36, 1:1,000 for WB), synapsin I (SySy, 106 011, 1:1,000 for WB, 1:500 for ICC), SALM4 (SySy, 294 403, 1:1,000 for WB), Myc (Cell Signalling, 2276S,1:1,000 for WB, not ICC), ERK (Cell Signalling, 9102S, 1:1,000 for WB), p38 (Cell Signalling, 9212S, 1:1,000 for WB), α-tubulin (Sigma, T5168, 1:10,000 for WB), NeuN (Millipore, MAP377, 1:300 for IHC), HA (Sigma, H6908/H3663, 1:500 for WB, 1:200 for ICC), Myc (Sigma, M4439, 1:500 for WB, 1:200 for ICC) and EGFP (Rockland,1:500 for ICC).

### Subcellular and PSD fractions

Subcellular fractionations of the rat brain were prepared as described previously^40^. PSD fractions were purified as described previously^41^.

### Mouse genotyping

Mouse genotyping was performed using the following three PCR primers: 5′-TCTCTGTAGCTCGTCTAGAC-3′ (5′ forward primer for both WT and KO alleles), 5′-CAGTCTATTACCATCTAGGTGC-3′ (3′ reverse primer for WT) and 5′-GTCTGTCCTAGCTTCCTCACT G-3′ (3′ reverse primer for KO). The PCR products for WT and KO alleles are 617 and 417 bp long, respectively.

### Immunohistochemistry and immunoblotting

Three-week-old mouse brain slices (50 μm) were permeabilized with 0.5% Triton X-100 in 1 × PBS for 1 h followed by primary and secondary antibody incubations. Fluorescent images were acquired using confocal laser scanning microscope (LSM510, Zeiss) and analysed using the MetaMorph software (series 7.1; Universal Imaging). We performed immunoblot experiments three times for Figs 3e and 6c and once for Figs 1b–f and 2d,i,j, Figs 4a–f and 5b–e and Fig. 7d, Supplementary Fig. 3e,f and Supplementary Fig. 6b.

### X-gal staining

Mice were transcardially perfused with heparinized 1 × PBS and 4% formaldehyde in 1 × PBS and postfixed at 4 °C for overnight. For X-gal staining, brain slices (250 μm) were sectioned using vibratome (Leica VT 1000S) and incubated in X-gal staining solution containing 1 mg ml^−1^ X-gal at 37 °C for overnight.

### Sholl analysis

Biocytin hydrochloride (0.5%; Sigma) was added to the pipette solution for whole-cell patch clamp, and infused into CA1 pyramidal cells, after measurements of basal transmission for general health check for 2–3 min. Then, the slices were fixed with 4% formaldehyde at 4 °C overnight and permeablized with 0.5% Triton X-100 in 1 × PBS. Alexa Fluor 555-conjugated streptavidin (2 mg ml^−1^, Invitrogen) in 1 × PBS was used for staining (1:300) for 1–2 h. Fluorescent images were acquired using confocal laser scanning microscope (LSM780, Zeiss).

### Electrophysiology

To measure mEPSCs and mIPSCs, CA1 pyramidal cells (P15–21, 250 μm with cold, oxygenated, 95% O_2_ and 5% CO_2_, sucrose-substituted ACSF (SCSF) (in mM): 212 sucrose, 10 glucose, 25 NaHCO_3_, 5 KCl, 1.25 NaH_2_PO_4_, 1.2 L-ascorbate, 2 pyruvate with 3.5 MgCl_2_ and 0.5 CaCl_2_) were held at −70 mV (6–8 min), using a MultiClamp 700B amplifier (Clampex 9.2, Molecular Devices). Pipette solutions contained (in mM) 117 CsMeSO_4_, 8 NaCl, 10 TEACl, 10 HEPES, 5 Qx-314Cl, 4 Mg-ATP, 0.3 Na-GTP and 10 EGTA (295 mOsm) for mEPSC measurements; or 115 CsCl, 10 TEACl, 8 NaCl, 10 HEPES, 5 Qx-314Cl, 4 Mg-ATP, 0.3 Na-GTP and 10 EGTA (294 mOsm) for mIPSC measurements. TTX (0.5 mM; Tocris) and picrotoxin (100 mM; Sigma-Aldrich) were added to oxygenated artificial cerebrospinal fluid (ACSF) (in mM): 125 NaCl, 10 glucose, 25 NaHCO_3_, 2.5 KCl, 1.25 NaH_2_PO_4_ with 1.3 MgCl_2_ and 2.5 CaCl_2_ for mEPSC recordings. TTX (0.5 mM), NBQX (10 mM; Tocris) and AP5 (25 mM; Tocris) were added for mIPSC measurements. Synaptic currents were analysed using a customized macro in Igor Pro 4.01 (WaveMetrics) and Clampfit (pCLAMP 10.4).

For field recordings, transverse hippocampal slices (400 μm) were prepared from 3- to 4-week-old male littermates. The brain was rapidly isolated and placed in ice-cold, oxygenated dissection buffer containing (in mM) 212.7 sucrose, 5 KCl, 1.23 NaH_2_PO_4_, 0.5 CaCl_2_, 10 MgCl_2_, 26 NaHCO_3_ and 10 glucose. Hippocampal slices were prepared by cutting with a Leica VT1000P vibratome (Leica) and transferred for recovery to a holding chamber containing oxygenated ACSF consisting of (in mM) 124 NaCl, 5 KCl, 1.23 NaH_2_PO_4_, 2.5 CaCl_2_, 1.5 MgCl_2_, 26 NaHCO_3_ and 10 glucose at 28–30 °C for at least 1 h before recording. After recovery, slices were transferred to a recording chamber where they were perfused continuously with oxygenated ACSF (27–28 °C) at a flow rate of 2 ml min^−1^. Hippocampal CA1 field excitatory postsynaptic potentials were evoked by Schaffer collateral stimulation (0.2 ms current pulses) with concentric bipolar electrode. Synaptic responses were recorded with ACSF-filled microelectrodes (1–2 MΩ) and were quantified as the initial slope of field excitatory postsynaptic potential). Data from slices with stable recordings (<5% change over the baseline period) were included in the analysis. All data are presented as mean±s.e.m. normalized to the preconditioning baseline (at least 20 min of stable responses). The experiments were blind to mouse genotypes. Recordings were performed using an AM-1800 Microelectrode amplifier (A-M systems), and the IGOR software (WaveMetrics, Lake Oswego, OR, USA) was used for digitizing and analysing the responses.

### Electron microscopy

*WT, Salm4*^−/−^ and *Salm3*^−/−^;*Salm4*^−/−^ mice were deeply anaesthetized with sodium pentobarbital (80 mg kg^−1^, intraperitoneally (i.p.)) and were intracardially perfused with 10 ml of heparinized normal saline, followed by 50 ml of a freshly prepared fixative of 2.5% glutaraldehyde and 1% paraformaldehyde in 0.1 m phosphate buffer (PB, pH 7.4). The hippocampus was removed from the whole brain, postfixed in the same fixative for 2 h and stored in PB (0.1 M, pH 7.4) overnight at 4 °C. Sections were cut transversely on a Vibratome at 70 μm. The sections were osmicated with 0.5% osmium tetroxide (in 0.1 m PB) for 1 h, dehydrated in graded alcohols, flat embedded in Durcupan ACM (Fluka) and cured for 48 h at 60 °C. Small pieces containing the stratum radiatum of hippocampal CA1 region were cut out of the wafers and glued on the plastic block by cyanoacrylate. Ultrathin sections were cut and mounted on Formvar-coated single-slot grids. For excitatory synapses, sections were stained with uranyl acetate and lead citrate, and examined with an electron microscope (Hitachi H-7500; Hitachi) at 80 kV accelerating voltage. For inhibitory synapses, sections were further immunogold-stained for GABA.

### Postembedding immunogold staining for GABA

Sections were immunostained for GABA by the postembedding immunogold method, as previously described^42^, with some modifications. In brief, the grids were treated for 5 min in 1% periodic acid to etch the resin, and for 8 min in 9% sodium periodate to remove the osmium tetroxide, and then washed in distilled water, transferred to Tris-buffered saline containing 0.1% Triton X-100 (TBST; pH 7.4) for 10 min and incubated in 2% human serum albumin in TBST for 10 min. The grids were then incubated with rabbit antiserum against GABA (GABA 990, 1:10,000) in TBST containing 2% human serum albumin for 2 h at room temperature. The antiserum (a kind gift from Professor O.P. Ottersen at the Center for Molecular Biology and Neuroscience, University of Oslo) was raised against GABA conjugated to bovine serum albumin with glutaraldehyde and formaldehyde^43^, and characterized by spot testing^44^. To eliminate cross-reactivity, the diluted antiserum was pre-adsorbed overnight with glutaraldehyde (G)-conjugated glutamate (500 μM, prepared according to a previous report^45^). After extensive rinsing in TBST, the grids were incubated for 3 h in goat anti-rabbit IgG coupled to 15 nm gold particles (1:25 in TBST containing 0.05% polyethylene glycol; BioCell Co., Cardiff, UK). After a rinse in distilled water, the grids were counterstained with uranyl acetate and lead citrate, and examined with an electron microscope (Hitachi H-7500; Hitachi) at 80 kV accelerating voltage. To assess the immunoreactivity for GABA, gold particle density (number of gold particles per μm^2^) of each GABA+ terminal was compared with gold particle density of terminals, which contain round vesicles and make asymmetric synaptic contact with dendritic spines (background density). Terminals were considered GABA-immunopositive (+) if the gold particle density over the vesicle-containing areas was at least five times higher than background density.

### Quantitative analysis of excitatory and inhibitory synapses

For quantification of excitatory synapse, 24 electron micrographs representing 368.9 μm^2^ neuropil regions in each mouse were taken at a × 40,000. Number of spines (PSD density), proportion of perforated spines, PSD length and PSD thickness from each three WT, *Salm4*^−/−^ and *Salm3*^−/−^*; Salm4*^−/−^ mice were quantified by using the ImageJ software. For quantification of inhibitory synapse, 24 electron micrographs representing 655.5 μm^2^ neuropil regions in each mouse were taken at a × 30,000. Number of GABA+ terminals showing clear PSD (inhibitory synapse density), length and thickness of PSD contacting GABA+ terminals from each three WT, *Salm4*^−/−^ and *Salm3*^−/−^*; Salm4*^−/−^ mice were quantified by using the ImageJ software. The measurements were performed by an experimenter blind to the genotype. Digital images were captured with the GATAN DigitalMicrograph software driving a charge-coupled device camera (SC1000 Orius, Gatan) and saved as TIFF files. Brightness and contrast of the images were adjusted in Adobe Photoshop 7.0 (Adobe Systems).

### Herpes simplex virus

Rat SALM4 in the pGW1-rSALM4 construct was subcloned into the p1005 HSV vector, a modified HSV amplicon plasmid^46^, to generate the HSV-SALM4 construct. For amplification and packaging of HSV-SALM4, or HSV-GFP (control), viruses, 2-2 cells (10% CO_2_) were transfected with HSV constructs and a helper virus. NIH3T3 cells were used to measure the titre of harvested viruses (HSV-EGFP: 1.9 × 10^8^ and HSV-SALM4: 1.2 × 10^8^ transducing unit ml^−1^). For HSV infection, anaesthetized mice head-fixed in the stereotaxic apparatus was bilaterally infused of HSV viruses (HSV-EGFP, 0.4 μl × 2 each hemisphere; HSV-SALM4, 0.6 μl × 2) at target regions (−1.3 AP/anteroposterior, ±1.4 medial lateral and −1.5 dorsal ventral) using a Hamilton syringe (World Precision Instruments Inc.) and 33-gauge blunt needle. For additional HSV experiments using HA-tagged SALM4 constructs, virus titres were 7.6 × 10^7^ transducing unit per ml for HSV-HA-SALM4 and 4.6 × 10^7^ for HSV-HA-SALM4-ecto. Injection conditions were as follows: HSV-EGFP (0.3 μl × 2 each hemisphere), HSV-HA-SALM4 (0.45 μl × 2 each hemisphere) and HSV-HA-SALM4-ecto (0.53 μl × 2 each hemisphere).

### Cultured neuron transfection and imaging

Cultured hippocampal neurons were prepared from E18 rat brains, as described previously^47^, on coverslips coated with poly-D-lysine and grown in Neurobasal medium supplemented with B-27 (Invitrogen), 0.5% fetal bovine serum, 0.5 mM Glutamax (Invitrogen) and sodium pyruvate (Invitrogen). For the overexpression of SALMs in cultured neurons, hippocampal neurons were transfected with pIRES-SALM2-EGFP, pGW1-Myc-SALM4 or EGFP (Control) using a CalPhos Kit (Clontech) at DIV10 and immunostained at DIV14. For the ICC, cultured neurons were fixed with 4% paraformaldehyde/4% sucrose, permeabilized with 0.2% Triton X-100 in PBS, immunostained with primary antibodies (against EGFP, Shank1 and SALM4 (to identify the triply transfected hippocampal neurons)), and Cy3-, Cy5- and fluorescein isothiocyanate-conjugated secondary antibodies (Jackson ImmunoResearch). The images were acquired using a confocal microscope (LSM710, Carl Zeiss) with a × 63 objective lens. All image settings were kept constant. The Z-stacked images were converted to maximal projection and analysed to obtain the size, intensity and density of the puncta immunoreactivities derived from marker proteins. The quantification was performed in a blind manner using MetaMorph (Molecular Devices).

### Mixed culture synapse-formation assays

Mixed culture assays were performed with HEK293T cells (American Type Culture Collection) as described^23^. Cultured hippocampal neurons were incubated until HEK293T cells transfected with EGFP (Control), or the indicated SALM3, SALM5 or LRRTM2 constructs, were added at 10 days *in vitro* (DIV 10) for further coculture experiments. HEK293T cells were transfected with FuGene (Roche, USA) as indicated. After 48 h, transfected HEK293T cells were trypsinized, seeded on the hippocampal neuron cultures at DIV 10, further cocultured for 48 h and double-immunostained with synapsin I, SALM4 and HA/GFP antibodies at DIV 12 as described previously^48^. All images were acquired using a confocal microscope. For quantifications, the contours of the transfected HEK293T cells were chosen as the region of interest. The fluorescence intensity of synapsin puncta normalized to each HEK293T cell area was quantified for red, green and blue channels with the MetaMorph Software (Molecular Device).

### Recombinant protein expression and purification

The Ig1-3 domains (aa 30–318) of human LAR-RPTP was cloned into the BamHI and XbaI sites of the pAcGP67 vector (BD Bioscience) tagged with the Fc domain of human IgG gene. The High Five insect cells (Invitrogen) were transfected with corresponding P4 baculovirus for 3 days and harvested. The supernatants containing secreted proteins were loaded on protein A-Sepharose column (GE Healthcare Life Science) for the purification of Fc-tagged proteins. The affinity resin was washed with the buffer containing 50 mM Tris-HCl (pH 8.0) and 200 mM NaCl. Fc-tagged proteins immobilized in the protein A-Sepharose column were eluted with glycine buffer (100 mM glycine, pH 2.1).

### Recombinant protein-binding assays

For recombinant LAR-binding assays, HEK293T cells expressing combinations of SALMs were incubated with extracellular solution containing (in mM):168 NaCl, 2.6 KCl, 10 HEPES, 2 CaCl_2_, 2 MgCl_2_, 10 D-glucose and 100 μg ml^−1^ BSA (pH 7.2) for 1 h at 4 °C, and then purified soluble LAR-Ig1-3-Fc proteins at 0.25, 0.5, 1, 1.5 and 2 mM concentrations were added and incubated for 1 h at 4 °C to allow protein binding; a single 2 mM was used for SALM5 binding. After washing of LAR-Ig1-3-Fc and fixation of the cells, LAR binding was visualized by incubating the cells with anti-human-Fc antibodies (Sigma; 2 μg μl^−1^, goat). Then, the cells were incubated with HA antibodies (Sigma; 1:500, mouse or rabbit), or Myc antibodies (Sigma; 1:500, mouse), without permeabilization for surface HA staining (HA-SALM3/CD8), followed by permeabilization and staining with SALM4 C-terminal antibodies (#2026; purified, 1:500, Gp).

### Mass spectrometric screen of SALM4-associated proteins

Preparation of protein samples, affinity chromatography and proteomic analysis were performed as described previously^49^. Briefly, crude synaptosomes were prepared from mouse forebrains (15 WT and KO mice; 3 weeks), and solubilized in 1 × PBS containing 0.5% Triton X-100, 0.1% SDS, 0.5 mM EDTA, 0.5 mM EGTA and protease inhibitor cocktail (Roche). From the solubilized proteins, mature membrane proteins were enriched using WGA column chromatography (WGA bead, Vector Laboratories) and eluted using N-acetyl-d-glucosamine (Sigma). The samples were incubated with SALM4 antibodies (2026, purified, Gp) prebound to protein A-Sepharose beads (GE Healthcare), followed by protein elution with 2.5 × XT MOPS buffer (BIO-RAD). Boiled samples were resolved using 4∼12% Criterion XT Precast Gels (BIO-RAD) followed by silver staining. From the stained gels, protein bands were excised and subjected to mass spectrometric analyses. Mass spectrometry was performed using LTQ-Orbitrap XL ETD (Thermo Scientific) at the Korea Basic Science Institute.

### Statistics

The results of statistical analysis are indicated in Supplementary Table 1.

### Data availability

The authors confirm that all relevant data are available from the authors.

## Additional information

**How to cite this article:** Lie, E. *et al*. SALM4 suppresses excitatory synapse development by *cis*-inhibiting *trans*-synaptic SALM3–LAR adhesion. *Nat. Commun.* 7:12328 doi: 10.1038/ncomms12328 (2016).

## Supplementary Material

Supplementary Information Supplementary Figures 1-6 and Supplementary Reference
