# Supplementary material for: SALM4 suppresses excitatory synapse development by cis-inhibiting trans-synaptic SALM3–LAR adhesion
Source: Nat Commun. 2016 Aug 2;7:12328. doi: 10.1038/ncomms12328 (PMC4974644; doi:10.1038/ncomms12328)
Supplement: Supplementary Information — Supplementary Figures 1-6 and Supplementary Reference [file ncomms12328-s1.pdf]

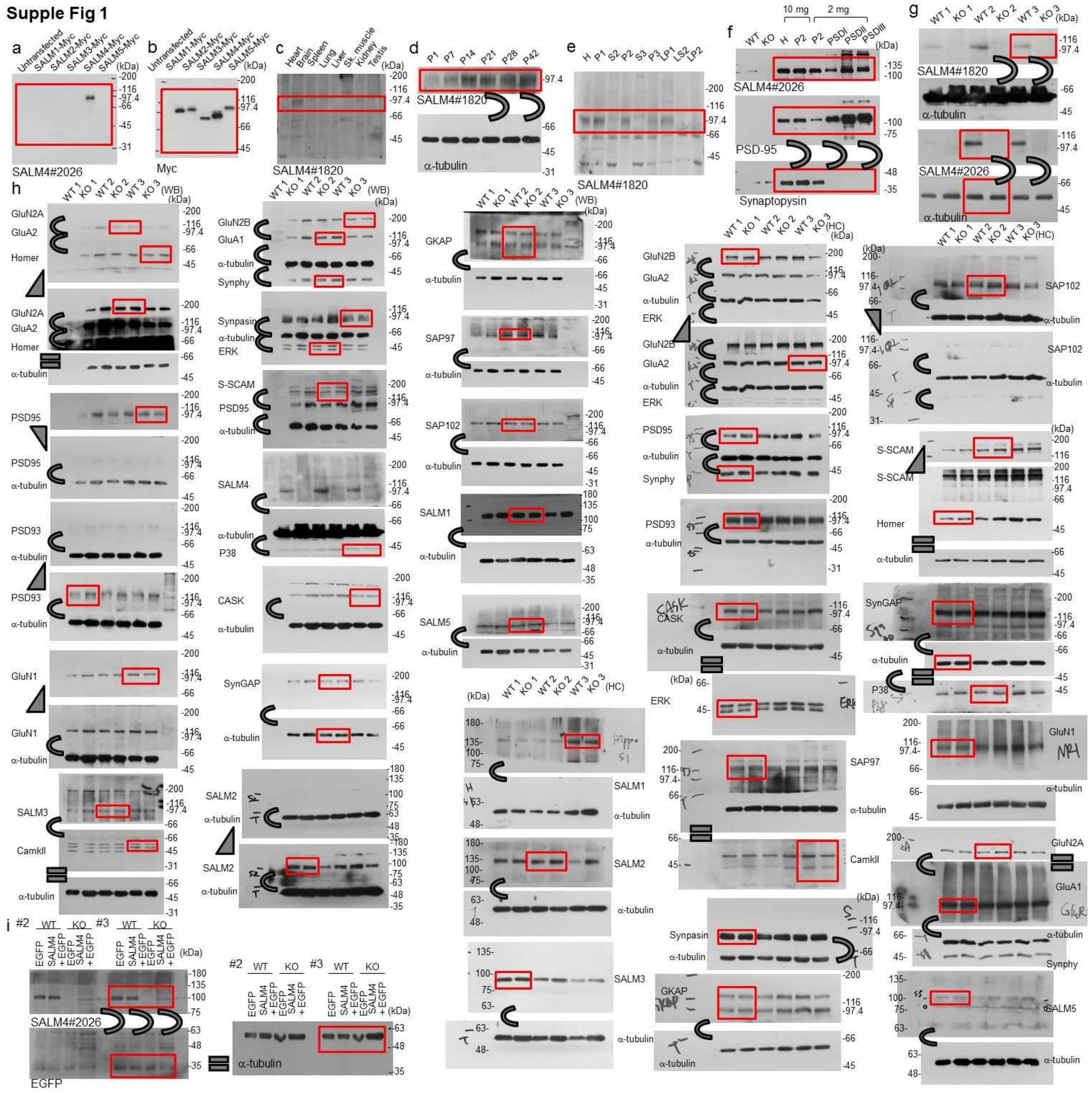

Supplementary Figure 1. Full-length immunoblots for main Fig 1-3.

(a) Full-length immunoblots for main Fig. 1b.

(b) Full-length immunoblots for main Fig. 1b

(c) Full-length immunoblots for main Fig. 1c

(d) Full-length immunoblots for main Fig. 1d. Note that the semi-circular connectors are linking two immunoblot membranes from the same gel.

(e) Full-length immunoblots for main Fig. 1e

(f) Full-length immunoblots for main Fig. 1f

(g) Full-length immunoblots for main Fig.2d

(h) Full-length immunoblots for main Fig. 2i,j. The triangle indicates different exposures of the same membrane, and the equal sign indicates stripping and re-blotting of the membrane with a different antibody.

(i) Full-length immunoblots for main Fig. 3e

Supple Fig 2

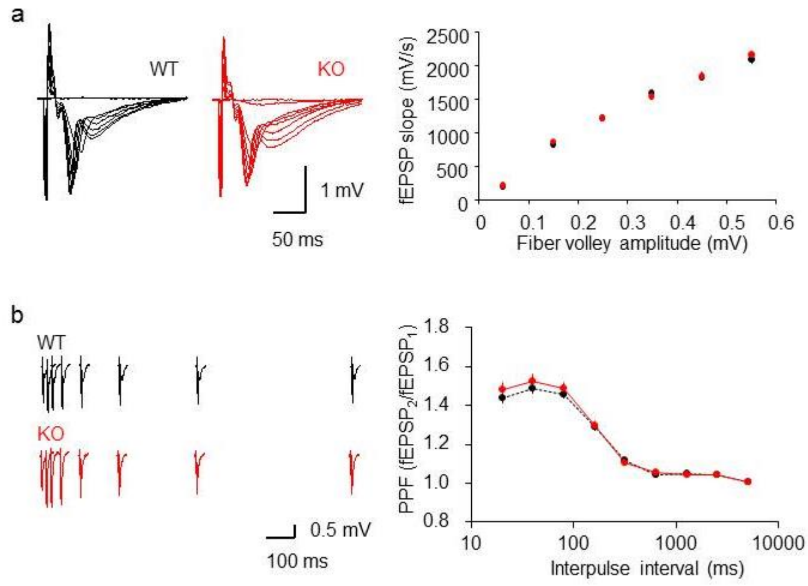

Supplementary Figure 2. Normal input-output relationship of evoked excitatory synaptic transmission and paired pulse facilitation at *Salm4*$^{-/-}$ Schaffer collateral-CA1 pyramidal synapses.

(a) Input-output relationship of evoked excitatory synaptic transmission, as indicated by fEPSP slopes plotted against fiber volley amplitudes. n = 20 slices from 11 mice for WT, and 23 (11) for *Salm4*$^{-/-}$.

(b) Paired pulse facilitation, as indicated by the ratio of consecutive fEPSP slopes ($fEPSP_2/fEPSP_1$) plotted against interpulse intervals. n = 31 slices (7 mice) for WT, and 30 (8) for *Salm4*$^{-/-}$.

Supple Fig 3

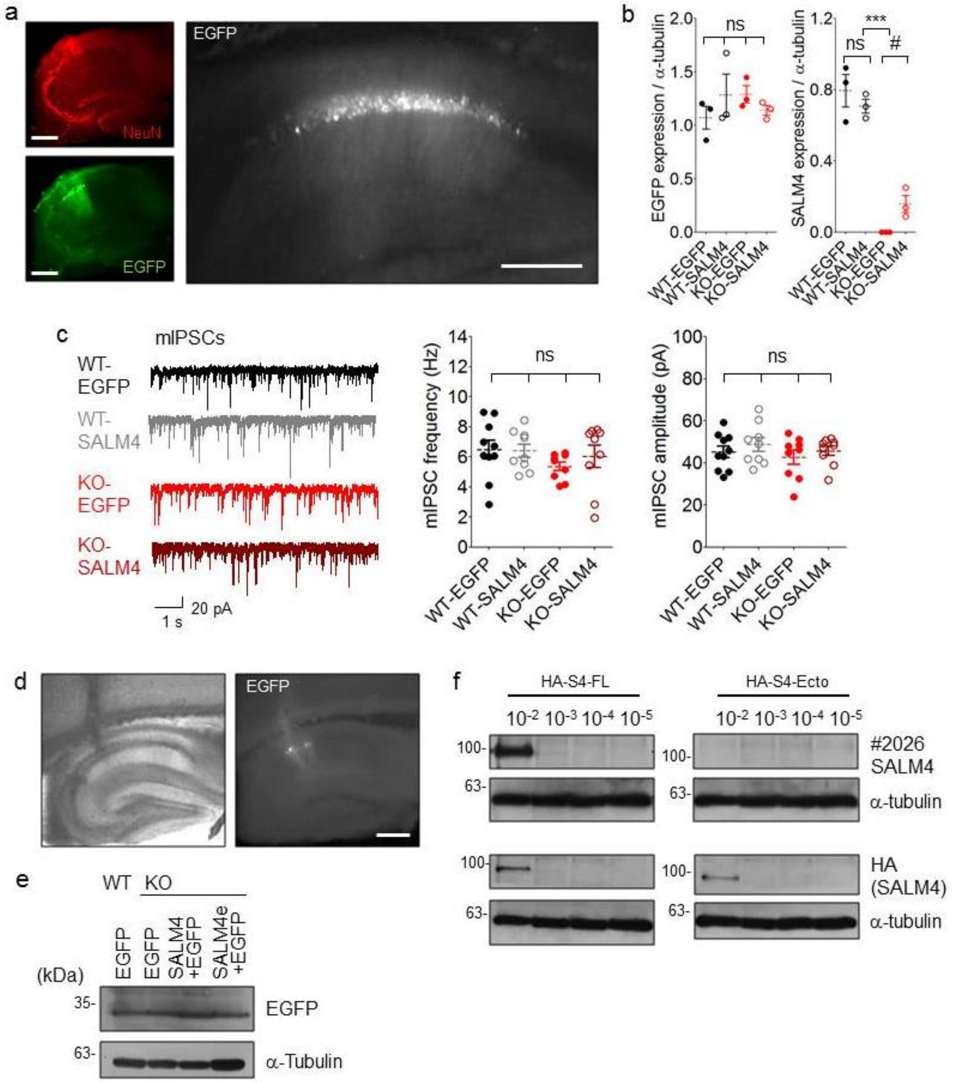

Supplementary Figure 3. HSV-mediated expression of SALM4 and variants in WT and *Salm4*$^{-/-}$ CA1 pyramidal neurons, and characterization of protein expression and mIPSCs.

(a) HSV-infected neurons expressing EGFP are indicated by EGFP fluorescence (black and white image), as well as by immunofluorescence imaging of EGFP in the background staining of NeuN (a neuron-specific marker). Scale bar, 500 μm.

(b) Quantification of the expression levels of EGFP and SALM4 proteins in infected slices. WT and *Salm4*$^{–/–}$ neurons in the mouse hippocampus were infected with HSV containing SALM4 + EGFP, or EGFP alone (postnatal day 15–18), and quantified by immunoblotting. Note that SALM4 levels in WT neurons in the presence and absence of SALM4 infection are not significantly different from each other likely due to the low number of infected cells. n = 3 slices, ***P < 0.001, ns, not significant, ANOVA, $^{\#}$P < 0.05, Student's t-test.

(c) mIPSCs measured from CA1 pyramidal neurons described in (b). n = 10 cells from 4 mice for WT-EGFP, 9 (3) for WT-SALM4, 9 (3) for KO-EGFP, and 9 (4) for KO-SALM4, ns, not significant (P = 0.4861 for frequency, P = 0.5462 for amplitude), ANOVA.

(d-f) Confirmation of EGFP expression in HSV-infected WT and *Salm4*$^{–/–}$ neurons. WT neurons were infected with HSV carrying EGFP, and *Salm4*$^{–/–}$ neurons were infected SSwith HSV carrying EGFP, EGFP + HA-SALM4, and EGFP + HA-SALM4-Ecto (P15–18), followed by immunofluorescence imaging of EGFP (d; an example from *Salm4*$^{–/–}$ neurons infected with HA-SALM4-Ecto) and immunoblotting for EGFP (e). Note that HA-SALM4 proteins were not detectable in these experiments likely due to the low titer of HSVs carrying HA-SALM4s relative to those containing untagged SALM4 (Fig. 3e), although we could detect SALM4 proteins from NIH3T3 cells, which are normally used to determine HSV titers (f). Note that SALM4-Ecto is not detected by the 2026 SALM4 antibody that was raised against the deleted C-terminal region of SALM4, whereas it is detected by the HA antibody targeting the N-terminal end of SALM4. Scale bar, 500 μm.

Supple Fig 4

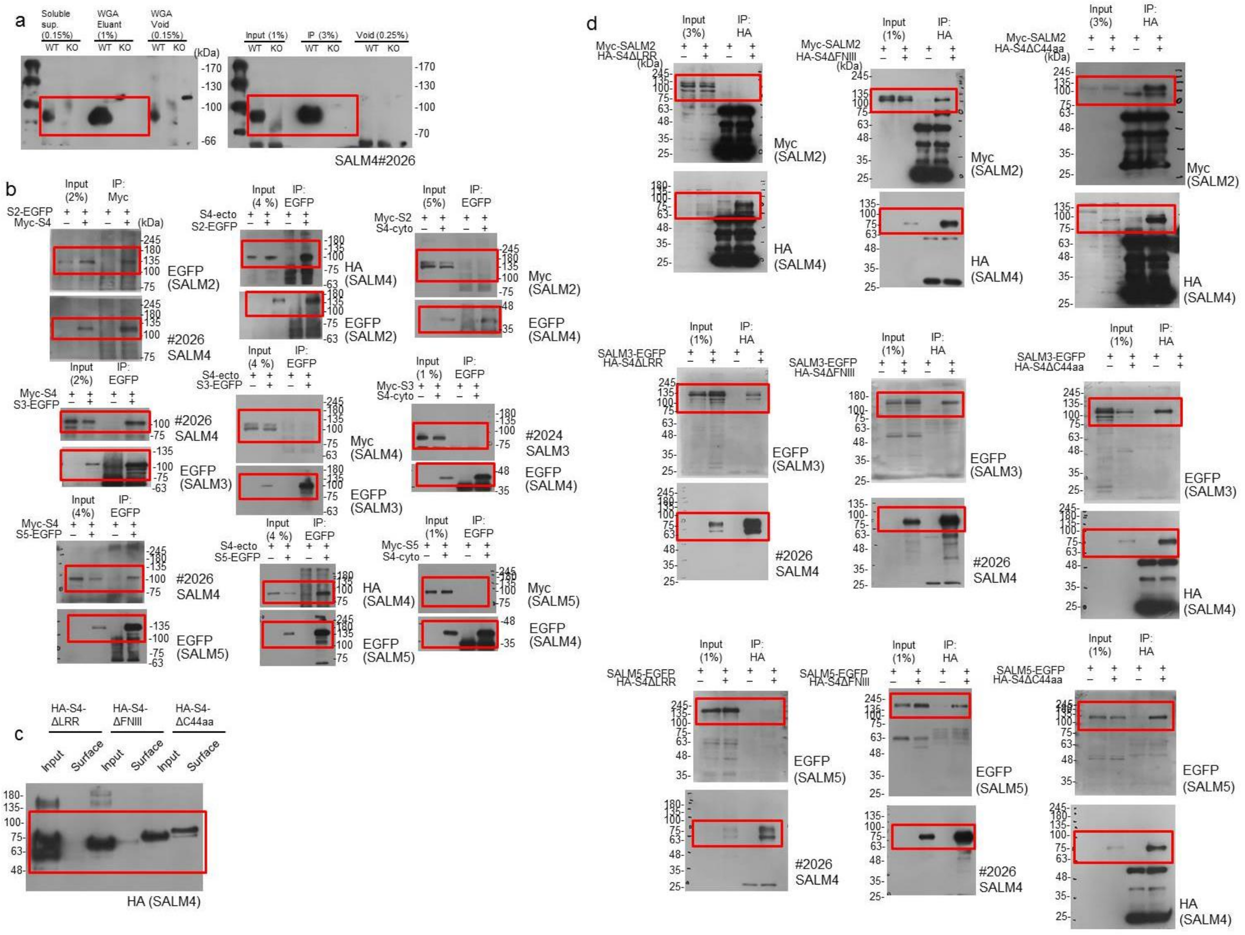

Supplementary Figure 4. Full-length immunoblots for main Fig 4-5.

(a) Full-length immunoblots for main Fig. 4a.

(b) Full-length immunoblots for main Fig. 4d-f. Note that the non-full-length blots for EGFP were caused by cropping of the membrane and were unavoidable in order to minimize excessive exposures of the antibody heavy and light chains used for coimmunoprecipitation that flank the EGFP band.

(c) Full-length immunoblots for main Fig. 5b.

(d) Full-length immunoblots for main Fig. 5c-e

Supple Fig 5

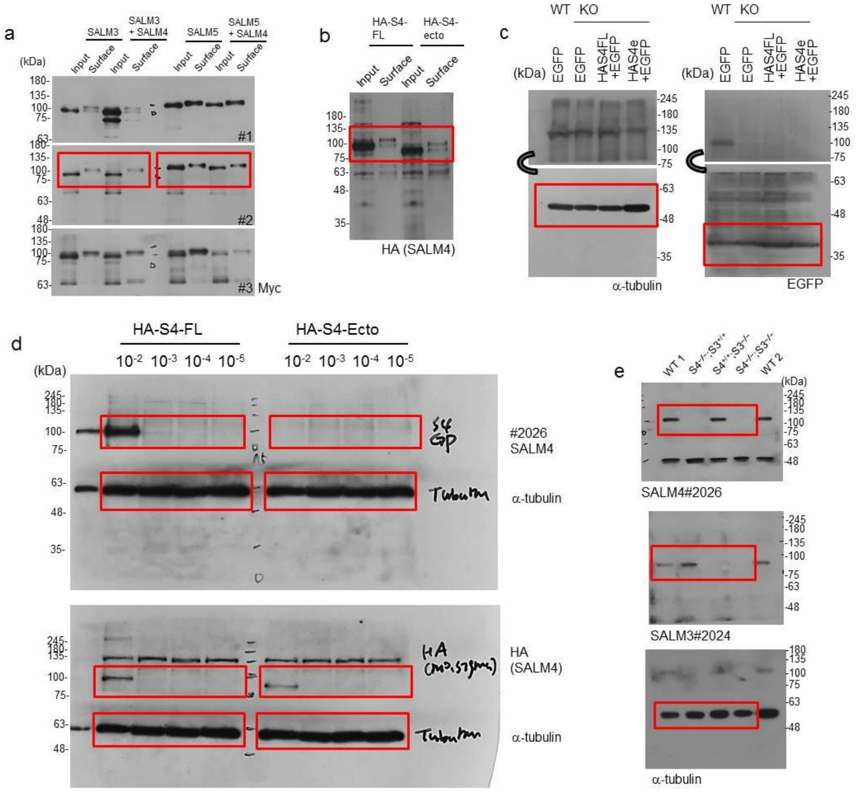

Supplementary Figure 5. Full-length immunoblots for main Fig 6 and 7 and supplementary Fig 3 and 6.

(a) Full-length immunoblots for main Fig. 6c.

(b) Full-length immunoblots for main Fig. 7d

(c) Full-length immunoblots for main Fig. Supplementary Fig. 3e.

(d) Full-length immunoblots for main Fig. Supplementary Fig. 3f.

(e) Full-length immunoblots for main Fig. Supplementary Fig. 6b.

Supple Fig 6

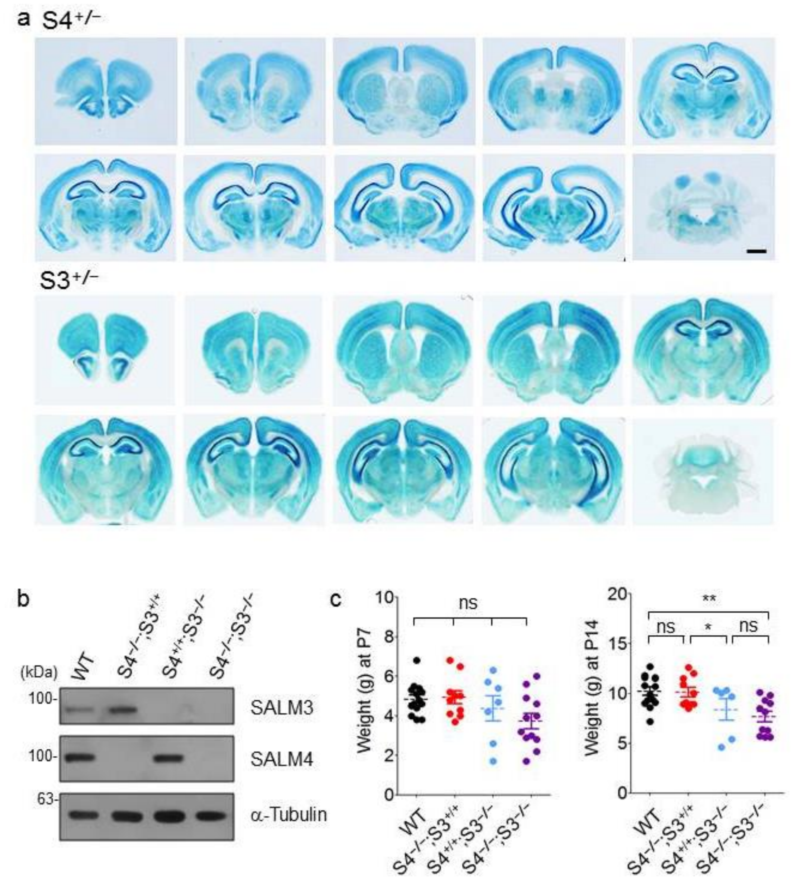

Supplementary Figure 6. SALM3 and SALM4 distribution patterns in the brain revealed by X-gal staining, and basic characterization of *Salm3*$^{-/-}$;*Salm4*$^{-/-}$ mice.

(a) Comparison of SALM3 and SALM4 protein expression patterns in the mouse brain, using X-gal staining of coronal *Salm3*$^{+/-}$ and *Salm4*$^{+/-}$ slices at 5 weeks. Note that the SALM3 and SALM4 signals detected in similar brain regions, including the cortex, hippocampus, and striatum. The X-gal images of *Salm3*$^{+/-}$ slices used here were taken from our previous publication for the purpose of comparison [1]. Scale bar, 1 mm.

(b) Whole brain lysates from the indicated single and double KO mice at P28 were immunoblotted for the indicated antibodies.

(c) The body weights of single and double KO mice were measured at P7 and P14. n = 14 for WT, 7 for *Salm3*$^{-/-}$, 10 for *Salm4*$^{-/-}$, and 12 for *Salm3*$^{-/-}$;*Salm4*$^{-/-}$ (P7), n = 14 for WT, 6 for *Salm3*$^{-/-}$, 10 for *Salm4*$^{-/-}$, and 11 for *Salm3*$^{-/-}$;*Salm4*$^{-/-}$ (P14), *P < 0.05, **P < 0.01, ns, not significant (P = 0.0688 for postnatal day 7), ANOVA.

Supplementary Reference

1. Li Y*, et al.* Splicing-Dependent Trans-synaptic SALM3-LAR-RPTP Interactions Regulate Excitatory Synapse Development and Locomotion. *Cell reports*, (2015).
